# Deciphering the temporal heterogeneity of cancer-associated fibroblast subpopulations in breast cancer

**DOI:** 10.1186/s13046-021-01944-4

**Published:** 2021-05-20

**Authors:** Freja Albjerg Venning, Kamilla Westarp Zornhagen, Lena Wullkopf, Jonas Sjölund, Carmen Rodriguez-Cupello, Pontus Kjellman, Mikkel Morsing, Morteza Chalabi Hajkarim, Kyoung Jae Won, Janine Terra Erler, Chris Denis Madsen

**Affiliations:** 1 Biotech Research and Innovation Centre (BRIC), University of Copenhagen (UCPH), Ole Maaløes Vej 5, 2200 Copenhagen N, Denmark; 2 Department of Laboratory Medicine, Division of Translational Cancer Research, Lund University, Scheelevägen 2, 22381 Lund, Sweden; 3 Novo Nordisk Foundation Center for Stem Cell Biology, DanStem, Faculty of Health and Medical Sciences, University of Copenhagen, 2200 Copenhagen N, Denmark

**Keywords:** Cancer-associated fibroblast (CAF), CAF heterogeneity, CAF subpopulations, Flow cytometry analysis, Breast cancer progression

## Abstract

**Background:**

Cancer-associated fibroblasts (CAFs) comprise a heterogeneous population of stromal cells within the tumour microenvironment. CAFs exhibit both tumour-promoting and tumour-suppressing functions, making them exciting targets for improving cancer treatments. Careful isolation, identification, and characterisation of CAF heterogeneity is thus necessary for ex vivo validation and future implementation of CAF-targeted strategies in cancer.

**Methods:**

Murine 4T1 (metastatic) and 4T07 (poorly/non-metastatic) orthotopic triple negative breast cancer tumours were collected after 7, 14, or 21 days. The tumours were analysed via flow cytometry for the simultaneous expression of six CAF markers: alpha smooth muscle actin (αSMA), fibroblast activation protein alpha (FAPα), platelet derived growth factor receptor alpha and beta (PDGFRα and PDGFRβ), CD26/DPP4 and podoplanin (PDPN). All non-CAFs were excluded from the analysis using a lineage marker cocktail (CD24, CD31, CD45, CD49f, EpCAM, LYVE-1, and TER-119). In total 128 murine tumours and 12 healthy mammary fat pads were analysed.

**Results:**

We have developed a multicolour flow cytometry strategy based on exclusion of non-CAFs and successfully employed this to explore the temporal heterogeneity of freshly isolated CAFs in the 4T1 and 4T07 mouse models of triple-negative breast cancer. Analysing 128 murine tumours, we identified 5–6 main CAF populations and numerous minor ones based on the analysis of αSMA, FAPα, PDGFRα, PDGFRβ, CD26, and PDPN. All markers showed temporal changes with a distinct switch from primarily PDGFRα+ fibroblasts in healthy mammary tissue to predominantly PDGFRβ+ CAFs in tumours. CD26+ CAFs emerged as a large novel subpopulation, only matched by FAPα+ CAFs in abundance.

**Conclusion:**

We demonstrate that multiple subpopulations of CAFs co-exist in murine triple negative breast cancer, and that the abundance and dynamics for each marker differ depending on tumour type and time. Our results form the foundation needed to isolate and characterise specific CAF populations, and ultimately provide an opportunity to therapeutically target specific CAF subpopulations.

**Supplementary Information:**

The online version contains supplementary material available at 10.1186/s13046-021-01944-4.

## Background

Cancer-associated fibroblasts (CAFs) contribute to a plethora of pro-tumorigenic functions, such as supporting cancer stem cells, providing protection against chemotherapy, and creating an immunosuppressive tumour microenvironment (TME) [1]. This has sparked great interest in targeting CAFs to treat various types of solid cancers [2]. While emerging data has questioned the long held belief that CAFs are genetically stable [3], CAFs are still promising drug targets that likely are less prone than cancer cells to develop mutational based resistance. On the other hand, resident fibroblasts within the tissue can present a barrier to oncogenic transformation of epithelial cells [4] and limit the proliferation of neoplastic cells [5], exemplifying desirable fibroblast functions. Emerging evidence also supports anti-tumorigenic potentials of CAFs [6, 7], adding a layer of complexity to CAF-targeting strategies. Which CAFs are tumour-promoting CAFs, and how can we distinguish these from the tumour-restraining CAFs? While studies reporting on tumour-suppressing functions of CAFs are clearly the minority, they nevertheless highlight the need for gaining more knowledge regarding CAF subpopulations with potentially opposite functions, if targeting of CAFs is to be a successful add-on to cancer treatment.

Intra-tumoural heterogeneity of cancer cells in breast cancer is well recognised, and emerging evidence points toward breast cancer CAFs being equally heterogeneous [8–18]. While cancer cell heterogeneity is believed to arise through clonal evolution, the origin(s) of CAFs, and how this influences CAF heterogeneity, are still being intensely studied. Evidence points towards multiple origins rather than one, with CAF-progenitor cells heralding from as diverse sources as adipose stem cells, endothelial cells, resident fibroblasts, bone marrow-derived mesenchymal stromal cells (BM-MSCs), and even from malignant cancer cells themselves [1, 2].

The implementation of single cell RNA sequencing (scRNA-seq) the last few years has reinforced the heterogeneous nature of CAFs in breast cancer, however the majority of the studies have not looked at the temporal and the spatial dynamics, nor have they correlated specific CAF populations to biological function. Among the few studies comparing functional differences are the findings that distinct CAF populations induce cancer cell migration through different mechanisms. The human CAF-S1 population (FAP^High^; CD29^Med-High^; αSMA^High^; PDPN^High^; PDGFRß^High^) promotes early epithelial-mesenchymal transition and secretion of factors that attract cancer cells, while the human CAF-S4 population (FAP^Low-Med^; CD29^High^; αSMA^High^; PDPN^Low^; PDGFRβ^Med^) promotes cancer cell invasion through matrix remodelling [14]. In addition, it has been shown that certain CAF populations, including the CAF-S1 population, exert immunosuppressive functions [10]. With the steady accumulation of scRNA-seq data, it is now clear that the CAF community needs to enter a next phase, where specific CAF populations need to be isolated for further characterisation of biological and molecular functions.

Though scRNA-seq clearly can identify different subpopulations of CAFs based on clustering of gene signatures, little information is presented at the protein level, and the ability to identify and isolate these subpopulations directly from fresh tissue. It is therefore important to identify cell surface receptors for isolating and purifying living subpopulations of CAFs for further ex vivo analyses. Moreover, information on the temporal development of different CAF populations during breast cancer and their correlation to the metastatic potential of the tumour cells is also lacking. To investigate these questions, we performed orthotopic implantation of syngeneic 4T1 or 4T07 cancer cells into the mammary fat pad of immune competent female mice. The two cell lines are both models of triple-negative breast cancer (TNBC) and originate from the same spontaneous breast tumour in a BALB/c mouse, but differ in their metastatic potential. 4T1 cells form primary tumours in syngeneic mice that readily spread and form macro-metastases in the lungs, liver, bone, and brain. 4T07 cells form primary tumours, but these tumours only manage to spread to the lymph nodes and to form micro-metastases in the lungs of syngeneic mice [19]. When isolating CAFs from different time points during tumour development, these two models offer the opportunity to study cancer cell intrinsic abilities to educate and recruit different CAF populations at different time points, in the setting of a fully functioning immune system.

Seeking to provide the CAF community with an unbiased starting point for isolating and investigating CAF heterogeneity in the context of any type of solid tumours in mouse cancer models, here we develop a multicolour flow cytometry (FCM) workflow, based on the principle of negative selection. By excluding essentially all other cell types in the TME, a CAF-enriched population is left behind ready for further ex vivo analysis. In this way, we purify living cells and avoid limiting the CAF population to predefined markers that are not all-encompassing. Our set-up also avoids ‘plastic-education’ typically observed when CAFs are isolated by letting the CAFs migrate out of a small piece of tumour to subsequently adhere to the culture dishes for days. As a proof of concept, here we investigate the co-expression of six CAF markers on primary breast cancer CAFs over time, and show how these markers define numerous specific CAF populations that change in abundance as the tumours grow.

## Material and methods

### Mouse model of TNBC

#### Breast cancer cell lines

The mouse breast cancer cell lines 4T1 (RRID:CVCL_0125) and 4T07 (RRID:CVCL_B383) are both derived from the same, spontaneous tumour from a BALB/c mouse [19]. Both cancer cell lines were acquired from the Karmanos Cancer Institute, USA, and cultured in DMEM w/L-Glutamine, high glucose and pyruvate, 10% FBS, 1% (100 U/ml) penicillin-streptomycin (Gibco, ThermoFisher Scientific, Copenhagen, Denmark) in a 37 °C humid incubator with 5% CO_2_. The cells were split 1:10 when they reached 70–80% confluency. Cell lines were routinely checked to be free of mycoplasma and cell line purity and identity was validated by short tandem repeats (STR)-profiling at the start of the project.

#### αSMA-RFP mice

To facilitate detection of the intracellular cytoskeletal protein αSMA in living cells such as CAFs, we used BALB/c mice genetically engineered to express a red fluorescent protein (RFP) simultaneously as the αSMA protein. These BALB/c αSMA-RFP transgenic mice express the fluorescent protein, DsRed-Express under the mouse *Acta2* gene promoter [20]. These mice were obtained from Raghu Kalluri, MD Anderson Cancer Center, USA and bred hemizygously in-house at our institute, but are now commercially available from The Jackson Laboratories (RRID:IMSR_JAX:031160).

#### Orthotopic breast tumour implantation model

When preparing cells for orthotopic implantation, the trypsinised cells were washed in PBS (Gibco, ThermoFisher Scientific, Copenhagen, Denmark) to remove remaining media and trypsin, and then re-suspended in PBS at 10^7^ cells/ml. The cell suspension was kept on ice until injection. Fifty μl cell suspension was injected into the lower left and right mammary fat pad of either 8–16 weeks old wild type BALB/c mice (for FCM control purposes) or hemizygous αSMA-RFP mice, using a 27G disposable needle, depositing 5 × 10^5^ cells per injection. Genetically identical cells, i.e. either 4T1 or 4T07, were implanted in both sides of each mouse in order to minimise the total number of mice. Tumour growth and animal welfare was monitored twice a week, following regulations stipulated by the Danish Animal Experiments Inspectorate. At 7, 14 or 21 days (D7, D14, D21) post injection the resulting primary tumours and remaining surrounding fat pad were collected in PBS on ice after euthanasia of the animals, and single cell suspensions prepared as described below.

#### Sample size

Three independent biological repeats of the orthotopic tumour models were carried out, and the sample size (number of tumours = technical repeats) within each biological repeat is listed below. Additionally, 12 healthy mammary fat pads were also collected and analysed in the same way as the tumour samples (Table 1).

**Table 1:**
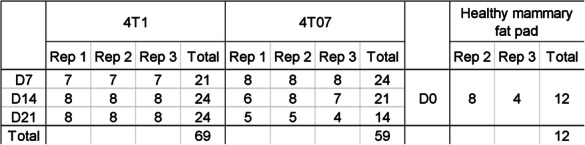
Tumour sample size in the study

Sample size was determined by the maximum number of samples it was possible to process in each biological repeat. Hemizygous αSMA-RFP mice were randomly allocated to be part of either the 4T1 or the 4T07 group, and injected with the respective tumour cells. On each collection day four animals from each tumour group were randomly selected for euthanasia and subsequent tumour collection. Due to paucity of cells in some tumour samples, the final number of tumours (technical repeats) analysed varies from 4 to the planned maximum of 8, with a total of 128 tumours analysed. The analysis of the tumour samples was not blinded.

### Flow cytometry

#### Dissociation of tumours into single cells

Tumours and cell suspensions were kept on ice between steps. Tissue was minced into roughly 2 × 2 mm pieces using disposable scalpels, and treated with the digestion enzyme mix from the mouse tumour dissociation kit by Miltenyi (Miltenyi Biotec Norden AB, Lund, Sweden, cat. # 130–096-730). Following the directions on the kit, the sample was then incubated in c-tubes (Miltenyi Biotec Norden AB, Lund, Sweden, cat. # 130–096-334) on the gentleMACS Octo tissue homogeniser w/ heaters (Miltenyi Biotec Norden AB, Lund, Sweden) to keep the mixture at 37 °C, using the pre-defined tumour_TDK2 program, running for 41 min. The sample was then washed with PBS and strained through a 70-μm mesh strainer to obtain a single cell suspension. Red blood cells (RBCs) were lysed using 1x RBC lysis solution from BD (Becton Dickinson Denmark A/S, Lyngby, Denmark, cat. # 555899), and cellular debris was removed according to directions in the Miltenyi Debris Removal Kit (Miltenyi Biotec Norden AB, Lund, Sweden, cat. # 130–109-398). The final single cell suspension was frozen in freezing media containing 50% DMEM 40% FBS and 10% DMSO, and kept frozen until the day of FCM analysis.

#### Sample preparation and antibody labelling

To minimize the technical noise and differences in antibody labelling, all frozen single cell suspensions of 4T1 and 4T07 tumours from a biological repeat were thawed and prepped for FCM analysis on the same day. For all washing steps and sample suspension, cold FACS buffer containing PBS + 2 mM EDTA + 1% BSA + 25 mM HEPES, pH 7 was used unless otherwise noted. Thawed samples were counted and a maximum of 10^7^ cells resuspended in 100 μl PBS and incubated on ice for 20 min with 1 μl Viobility-405/520 amine reactive viability dye (Miltenyi Biotec Norden AB, Lund, Sweden, cat. # 130–110-206) per 100 μl cell suspension. Excess viability dye was washed off using FACS buffer, and samples were incubated in 200 μl biotin labelled lineage marker antibody cocktail for 30 min at 4 °C followed by washing in FACS buffer. Lastly, samples were incubated 30 min in the dark at 4 °C in 100 μl CAF marker antibody cocktail per 2 × 10^6^ cells, then washed 3 times in FACS buffer and kept in the dark on ice until acquisition on the BD LSRII flow cytometer. The CAF marker antibody cocktail included the streptavidin-BV711 to simultaneously detect the biotin-labelled lineage positive cells.

### Reagents and multicolour FCM panel

#### Lineage marker cocktail

The lineage cocktail (Table 2) consisted of biotin labelled, primary antibodies detecting endothelial cells (CD31), immune cells (CD45), basal myoepithelial cells of the mammary ducts (CD49f) [21, 22], lymphatic endothelial cells (LYVE-1), and erythroid lineage cells (TER-119). Cancer cells were excluded using EpCAM/CD236, CD24 and CD49f. A CAF marker antibody cocktail (Table 3) was prepared to detect the 6 chosen CAF markers; fibroblast activation protein alpha (FAPα), platelet derived growth factor receptor alpha and beta (PDGFRα and PDGFRβ), and CD26/DPP4 and podoplanin (PDPN), and alpha smooth muscle actin (αSMA).

**Table 2:**
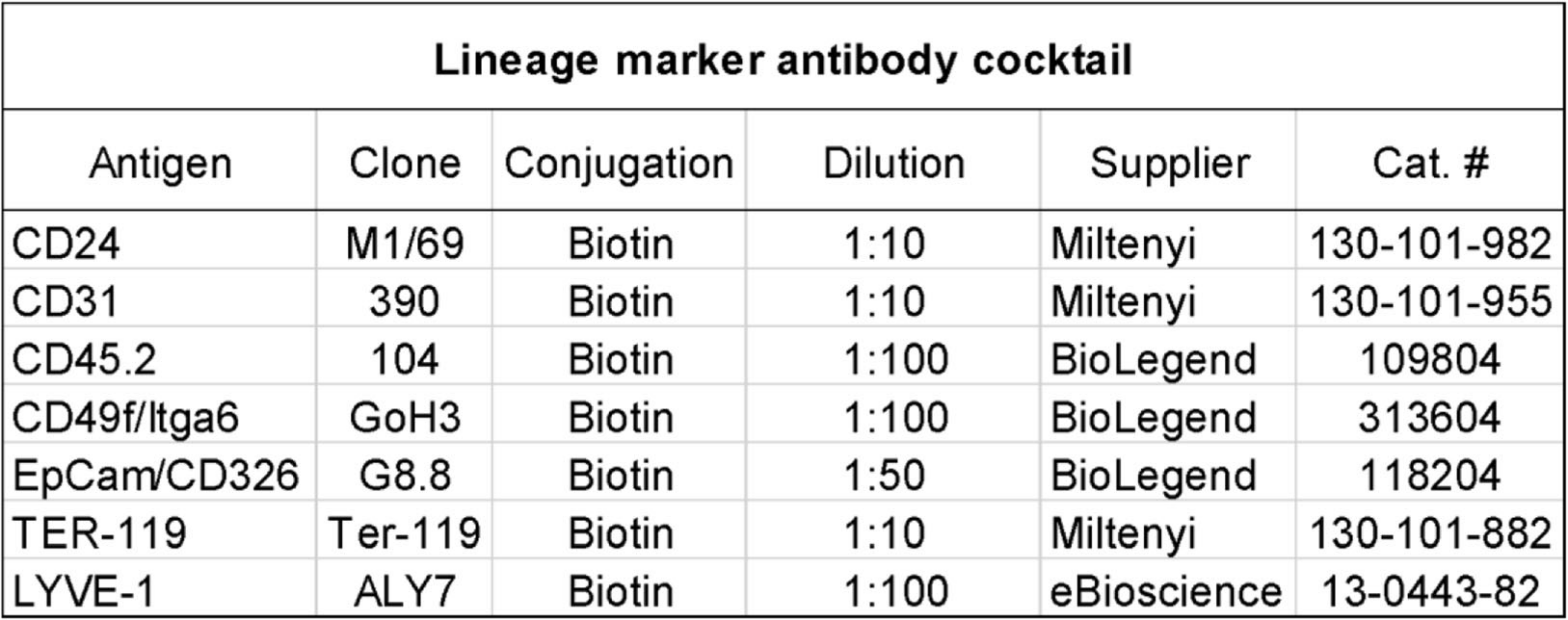
Lineage marker antibody cocktail

**Table 3:**
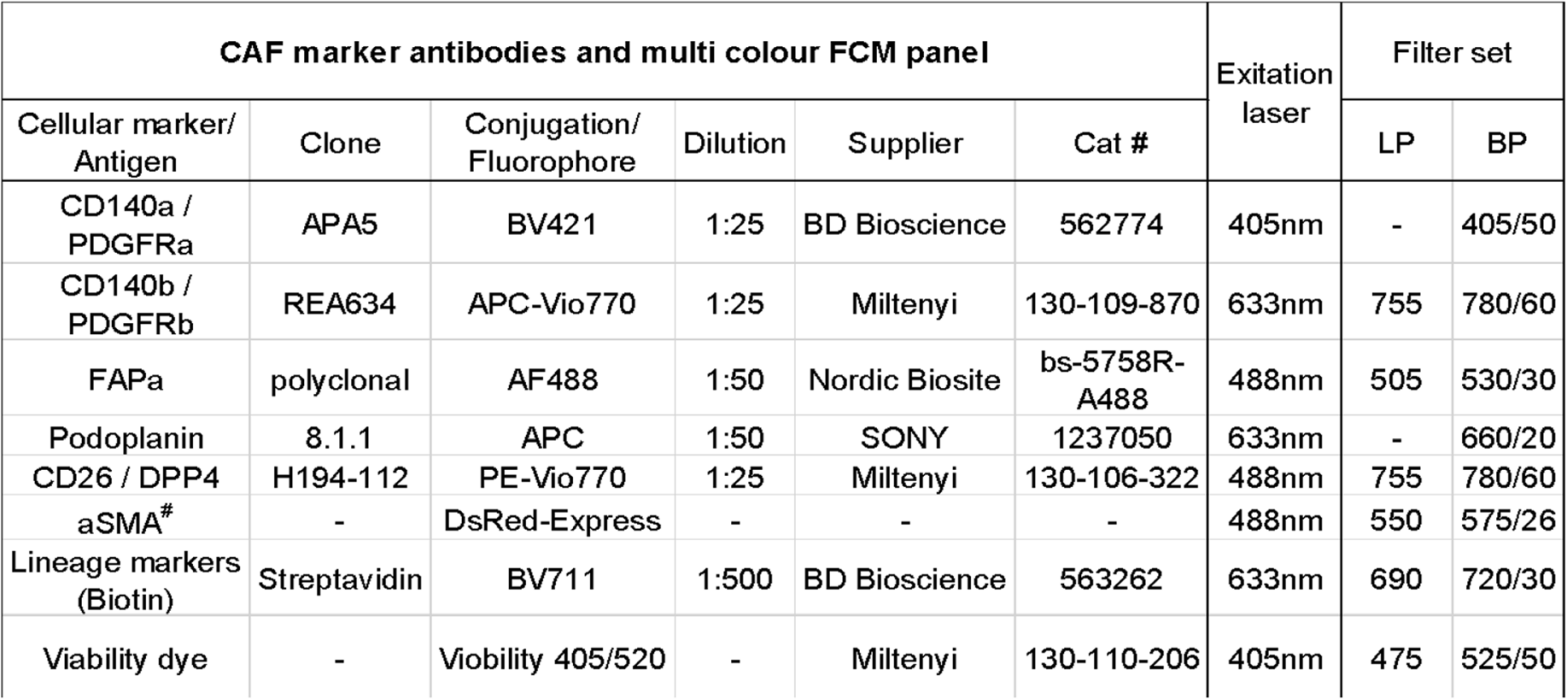
CAF marker antibody cocktail

^#^ DsRed is ectopically expressed in cells also expressing αSMA, no antibody needed

The Streptavidin is included in this table as it was admixed with the CAF marker antibodies to a final cocktail applied to the samples after incubation with the lineage marker cocktail. *LP* long pass filter, *BP* band pass filter

### Data acquisition

All FCM data were acquired on a BD LSRII analyser using DIVA software. The fluorescent antibodies on the cells were excited by either a 405 nm, 488 nm or a 633 nm laser in the LSRII analyser. BD cytometer setup and tracking (CS&T) beads were run daily on the analyser to calibrate the laser setup. The PMT voltages were checked before each run, and adjusted if necessary to assure that the positive population of the single stained controls were within detection range.

### Compensation controls

For compensation purposes, at least 5000 positive events were acquired from single colour controls, one for each fluorophore. These single stained controls were prepared by adding the fluorophore labelled antibodies to a drop of UltraComp Beads (eBioscience, ThermoFisher Scientific, Copenhagen, Denmark, cat # 01–2222-42), followed by 30 min incubation in the dark at 4 °C, and subsequent washing with FACS buffer. An exception to this was the CD140b-APC-Vio770 antibody, which does not bind to the UltraComp beads, and thus here an aliquot of single cell suspension from a tumour grown in a wild type (RFP^−/−^) mouse was used instead of the beads. Fibroblasts isolated from a healthy hemizygous αSMA-RFP mouse were grown on plastic to induce αSMA expression, and used as single colour control for the DsRed fluorophore. A sample of a tumour grown in a wild type (RFP^−/−^) mouse was used as a negative control for the APC-Vio770 antibody and the DsRed fluorophore, while the other single colour controls on the UltraComp beads carried their own internal negative control in the form of beads not binding to any antibodies. A compensation matrix was created based on the compensation controls, applied to the FCS files in the FCS Express 6 software, and compensated FCS files were used for all subsequent gating and analysis.

### FCM data analysis

All FCS files were analysed using the FCS Express 6 software.

### Gating controls

No isotype controls were used in this study as this type of control has very limited usage in helping proper gating in multicolour FCM, where most of the background is due to spill-over from fluorophores with overlapping spectra [23]. Fluorescent minus one (FMO) controls were used for each fluorophore to help guide proper gating on each marker [24] (see Suppl. Fig. 1). The FMOs were prepared by staining tumour samples with a mix of all the conjugated-antibodies within the panel except one antibody, ending up with multiple FMO tubes, each tube lacking a specific fluorophore. The gates for each of the CAF markers were set so no more than 0.03% of the events in the FMO control sample fell within the gate.

### Gating strategy

**Fig. 1:**
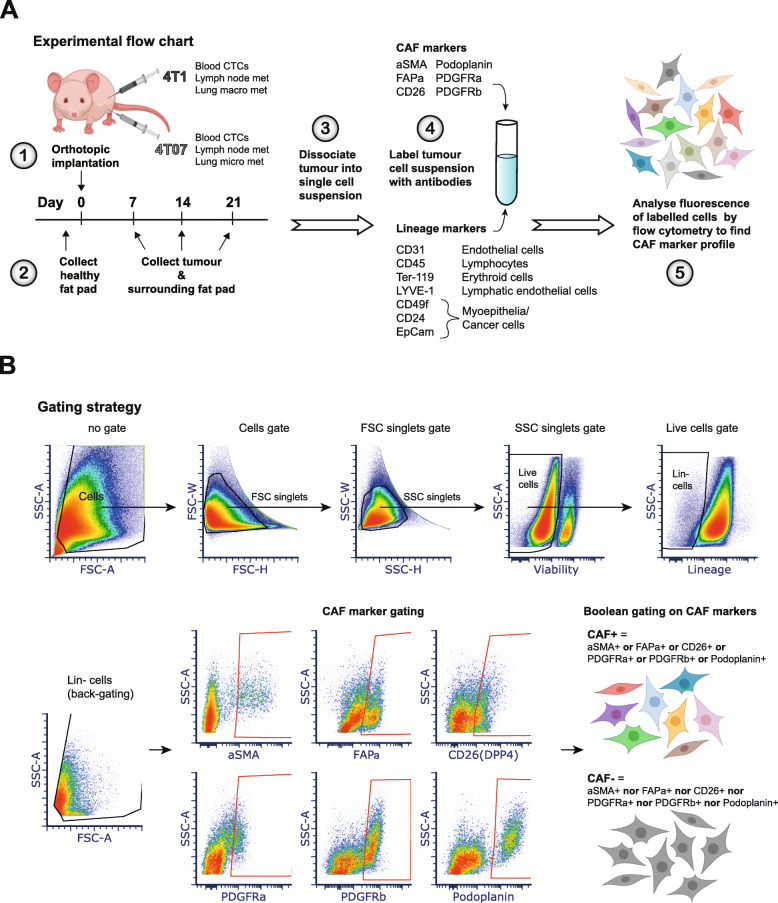
Experimental set-up and gating strategy for flow cytometry analysis. **a** Schematics of experimental setup. 4T1 or 4T07 breast cancer cells were injected into the mammary fat pad of αSMA-RFP mice. 4T1 and 4T07 are sister cell lines derived from the same tumour, but have different metastatic profiles as denoted next to them in the schematic. CTCs = circulating tumour cells, met = metastases. **b** Flow cytometry gating strategy to obtain live cells devoid of lineage markers (Lin-), and subsequent gating on each CAF marker used for Boolean gating to define the CAF- and CAF+ populations. The gates for all markers were set using the fluorescent minus one (FMO) approach. The density plots show one representative 4T1 D7 tumour (D7: collected at day 7). FSC = forward scatter, SSC = side scatter

Cells were gated on forward and side scatter (FSC and SSC), and doublets were excluded by both FSC-H vs FSC-W and SSC-H vs SSC-W bivariate plots. Live cells were gated as Viobility 405/20- cells, and Lineage negative (Lin-) cells were gated on Live cells as BV711- cells (EpCAM-CD45-CD31-TERT119-CD24-CD49f- and LYVE-1-). CAF+ cells were then gated on Lin- cells as cells positive for at least one of the 6 CAF markers using Boolean criteria (αSMA+ OR FAPα+ OR CD26+ OR PDGFRα+ OR PDGFRβ+ OR PDPN+). See Fig. 1b for visual representation of gating strategy.

### FCM data visualisation

Percentages from Boolean gating on the six CAF markers performed in FCS express 6 was exported to an excel spreadsheet and visualised using the freeware SPICE (https://niaid.github.io/spice/) [25]. Each of the possible Boolean combinations (Boolean CAF subpopulations) were plotted as slices in a pie chart, with the arcs around the pie charts corresponding to one CAF marker. All samples within a group defined by tumour and day, e.g. 4T1 D7, were grouped together and averaged (relative scaling), so the pie slices show the mean percentage of each CAF subpopulation within the total CAF+ population. The heat-map over the ‘Boolean CAF subpopulation abundance’ across time and tumour type was created using the clustering freeware TreeView3.0 (https://bitbucket.org/TreeView3Dev/treeview3/src/master/). All bar plots, graphs, and box and whisker plots were created using Graph Pad Prism version 6.

### UMAP analysis

The intensity levels of each of the six CAF markers were normalised into a range from 0 = no signal to 1 = strongest signal. The UMAP dimension reduction of the Lin- population was performed as previously described (https://arxiv.org/abs/1802.03426) [26].

### Statistics

All statistical tests were performed using Graph Pad Prism version 6. Parametric test were used and deemed appropriate due to the principle of the central limit theorem. The central limit theorem states that given a sufficiently large sample size, the sampling distribution of the mean for a variable approximates a normal distribution, regardless of that variable’s distribution in the population that is being sampled. This means that for large datasets the central limit theorem ensures that it is appropriate to use parametric tests, since the sampling distribution approximates a normal (Gaussian) distribution. The authors estimated that the size of the dataset in this study meant that the central limit theorem would make parametric tests appropriate, even if the data deviate slightly from a normal distribution. When comparing CAF populations across time within each tumour type (intra-tumour time differences) 2-way ANOVA was used to analyse the dataset, followed by Tukey’s multiple comparisons post-test with statistical significance set to α = 0,05. In case the 2-way ANOVA found tumour type not to be a significant variable, all tumours were pooled and analysed by one-way ANOVA to assess the impact of time and test for a possible linear trend. Multiple unpaired, two-tailed t-tests without assuming equal variance were used to compare time points across tumour type (inter-tumour differences), using the false discovery rate (FDR) approach with false discovery rate Q = 1% to correct for multiple comparisons. When comparing pie charts generated in SPICE a build-in permutation test was used. The permutation test asks how often, given the samples that comprise the compared pies, the difference observed would happen simply by chance (https://niaid.github.io/spice/help/analysis-comparingoverlays). Detailed statistical analyses are shown in Supplementary Tables 1, 2, 3, 4, 5, 6, 7, 8, 9, and 10.

### Single cell RNA-Seq analysis

The mammary gland Tabula Muris droplet data [27], was downloaded from https://github.com/czbiohub/tabula-muris. The two human breast cancer datasets [15, 18], were downloaded from Broad Institute’s Single Cell Portal https://singlecell.broadinstitute.org/single_cell and from https://blueprint.lambrechtslab.org. Matrices quality control filtered by the authors were used for the analysis. The datasets were then normalized and scaled using the basic pipeline of Seurat (version 4.0.0, https://www.biorxiv.org/content/10.1101/2020.10.12.335331v1). Clustering was performed with default parameters. Cell-type annotations in all three datasets were obtained by the authors. The gene signature (Acta2, Fap, Pdgfra, Pdgfrb, Dpp4 and Pdpn) was quantified as the average Z-score of member genes and applied to the three single cell datasets.

## Results

### Unbiased detection of CAFs through negative selection strategy

We carried out three independent biological repeats of the orthotopic breast tumour model shown in Fig. 1a, implanting either 5 × 10^5^ 4T1 or 4T07 cells into the mammary fat pad of the αSMA-RFP BALB/c mice, analysing a total of 128 tumours and 12 healthy mammary fad pads. We decided to employ a negative selection strategy in order not to limit our dataset to one specific CAF marker, giving us an unrestricted starting point for investigating CAFs in 4T1 and 4T07 tumours. We designed an antibody cocktail of lineage markers (CD24, CD31, CD45.2, CD49f, EpCAM, TER-119 and LYVE-1) to exclude non-fibroblast cells from the single cell suspension of tumours analysed by flow cytometry (FCM) (Fig. 1a and Table 2). To exclude cancer cells we initially used GFP labelled cancer cells, however pilot experiments suggested that GFP expression was either lost or greatly diminished from the tumours around 14 days after implantation (data not shown), prompting us to choose another way of excluding the cancer cells. The expression of EpCAM, CD24, and CD49f have been used to detect different subtypes of breast cancer cells [28], thus we tested the expression of these markers in the 4T1 and 4T07 cell lines (Suppl. Fig. 2). The metastatic 4T1 cell line expressed all three markers, while the less aggressive 4T07 only expressed CD24 and CD49f. As breast cancer cell lines also exist in heterogeneous states [28], we decided to include all three markers in our lineage cocktail to exclude as many cancer cells as possible. The population of sorted live cells that did not express any of the lineage markers was termed lineage negative (Lin-) (Fig. 1b).

### Fibroblast population size depends on time and tumour type

**Fig. 2:**
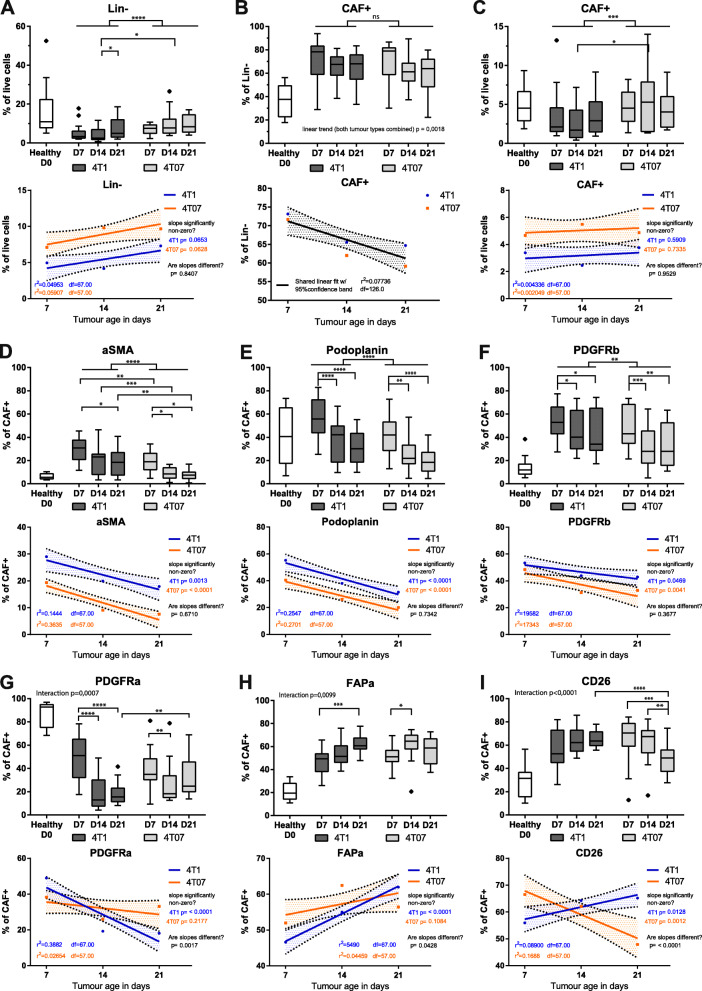
Analysis of 128 tumours reveal temporal dynamics and an effect of tumour type on population size. **a-c** Percentage of cells in healthy mammary fat pad (D0) and 4T1 and 4T07 tumours across the different time points. **d-i** Percentage of cells in healthy mammary fat pad (D0) and 4T1 and 4T07 tumours across the different time points positive for the respective CAF marker within the CAF+ population. Tukey box plots of two (healthy samples) or three independent repeats (tumour samples) combined. The boundaries of the Tukey style box go from the 25th to the 75th percentile, and the median is depicted by a line. The healthy samples provide a D0 reference point, but all the statistics are only concerning the tumour samples. Fitting a straight line to the tumour data, testing if one line fits both 4T1 and 4T07 tumours to indicate no difference, or if slope and/or y-intercept differ if two lines better capture the dataset. Fitted lines are shown with hashed out 95% confidence bands (CB95), with dots indicating the observed mean at the respective time points. **a-c** To determine statistical significance 2-way ANOVA with Tukey’s multiple comparisons post-test was used for intra-tumour comparisons, and multiple, two-tailed, t-tests with FDR correction (Q = 1%) were used for inter-tumour comparisons. **d-i** To determine statistical significance between time points within each tumour type (intra-tumour time comparisons) a 2-way ANOVA with Tukey’s multiple comparisons post-test was run on all the CAF markers combined, once for 4T1 tumours and once for 4T07 tumours. To determine if markers differed between tumour types, multiple unpaired, two-tailed t-tests without assuming equal variance and with FDR correction (Q = 1%) were conducted for each of the three time points (inter-tumour comparison).* = *p* < 0.05, ** = *p* < 0.01, *** = *p* < 0.001, **** *p* < 0.0001. Healthy *n* = 12, 4 T1 D7 *n* = 21, 4 T1 D14 *n* = 24, 4 T1 D21 *n* = 24, 4 T07 D7 *n* = 24, 4 T07 *n* = 21, 4 T07 D21 *n* = 14

Most reports point towards CAFs having a tumour supportive role, regardless of the CAF marker chosen to represent the CAF population. Thus, the initial question we sought to answer was what percentages of live cells in the TME are CAFs? Due to our negative selection strategy, the Lin- population is highly enriched for fibroblasts and CAFs, and we investigated whether this population differed in size during tumour progression and between the two tumour types. We first evaluated the percentage of purified Lin- cells in healthy mammary fat pad and observed an average of ~ 10% Lin- cells in 8–16 weeks old mice without tumours. Not surprisingly, this percentage decreased as the tumours developed (day 7 (D7), day 14 (D14), day 21 (D21)), simply because the Lin- cells were being outnumbered by the expanding and proliferative tumour cells in the mammary fat pad (Fig. 2a). We then examined if the percentage of Lin- cells increased as the tumour grew. These observations demonstrate that the two tumour types have different relative levels of Lin- cells, but no statistically significant change in the relative size of the Lin- population was observed (Fig. 2a). Interestingly, 4T07 tumours had a significantly higher percentage of Lin- cells as compared to 4T1 tumours, irrespective of tumour age (Fig. 2a. and Suppl. Fig. 2).

### Six CAF markers combined capture the majority of Lin- cells

A multitude of markers have been proposed as CAF markers, including but not limited to: αSMA, FAPα, fibroblast specific protein-1 (FSP1/S100A4), vimentin, PDPN, PDGFRα, PDGFRβ, CD90 (Thy.1), neuron glial antigen 2 (NG2), Caveolin-1 [29], and recently α11-integrin [30, 31]. In reality, each marker defines separate cell populations with partially overlapping molecular traits, and no single marker can therefore define the full CAF population, nor distinguish them from other cell types. It therefore remains unclear to what extend these markers truly represent the total level of CAFs within the TME. To allow for future FACS-based isolation and subsequent live-cell studies we focused our analysis on cell-surface markers. We therefore took advantage of the αSMA-RFP mouse that expresses red fluorescent protein (RFP) under the alpha smooth muscle actin (αSMA) promotor [20], considered to be the bona fide marker of activated myo-fibroblasts and myofibroblastic CAFs.

We first performed a bioinformatics analysis of well-known cell surface markers of CAFs from three different scRNA-seq datasets. First, we looked at cell surface markers in the Tabula Muris compendium containing nearly 100,000 cells from 20 organs and tissues from *Mus musculus*. Uniform Manifold Approximation and Projection (UMAP) of the different cell population demonstrated that FAPα, PDGFRα, PDGFRβ, and PDPN clustered to stromal cells of the mammary gland, while the αSMA+ cells as expected, clustered to pericytes and basal epithelial cells (Suppl. Fig. 3). We also examined the gene dipeptidyl petidease-4 (DPP4/CD26), a close homologue to FAPα [32], which was recently identified as a fibroblast marker of interlobular fibroblasts in healthy breast tissue and shown to display immune suppressive functions in breast cancer [10, 33]. The expression of CD26 likewise clustered to stromal cells in the healthy murine mammary gland. Finally, we examined the S100A4 gene, but as demonstrated by the UMAPs this gene was expressed by different cell types and in particular in macrophages, and we therefore decided to exclude this marker from future analyses. When simultaneously overlaying the expression of FAPα, PDGFRβ, PDGFRβ, PDPN, αSMA and CD26 onto the Tabula Muris UMAPs, these markers nicely associated with stromal fibroblasts in the health mammary gland. We then examined these six markers in two scRNA-seq studies from human breast cancers and demonstrated that all 6 genes effectively clustered to 1) the fibroblast cluster identified in 14 treatment-naïve breast cancers [15], and 2) the four stromal subpopulations identified in 5 patients with TNBC [18]. These analyses demonstrate that all six markers identify CAFs in breast cancer, and we therefore designed an antibody panel (see Table 3) consisting of the 5 cell surface CAF markers: FAPα, PDGFRα, PDGFRβ, PDPN and CD26.

To simplify the analysis, we chose to make the presence of each CAF marker a binary question, assigning each cell to either the positive or negative population based on fluorescent minus one (FMO) gating. We did this for each of the 6 markers, and using Boolean criteria we could define two Lin- populations: a) Lin- cells that do not express any of the 6 selection markers (we termed this population CAF-) and b) Lin- cells that express at least one of the 6 markers (we termed this population CAF+) (Fig. 1b). As anticipated, our experimental setup with 6 CAF markers did not lead to full coverage of the Lin- population, although still capturing 60–80% of the Lin- cells in tumours (Fig. 2b). The analysis also demonstrated that the percentage of CAF+ cells in relation to live cells was relatively stable over time in tumours (Fig. 2c, lower panel). Interestingly, the overall percentage of CAF+ cells in relation to Lin- cells was actually decreasing during cancer development regardless of tumour type (Fig. 2b, linear trend *p* = 0,0018), meaning that the CAF- population, not expressing any of the 6 markers, was increasing. The CAF- population may therefore represent other CAF subpopulations expressing markers not used in this panel. In summary, our workflow using 5 well-established markers and one new CAF marker (CD26) captured 60–80% of all Lin- cells in the primary tumours, and it is thus reasonable to assume that our workflow captured the majority of bona fide CAFs.

### CAF marker prevalence depends on tumour age and type

We then sought to explore how each individual CAF marker was temporally present in each tumour type. The first observation was a dramatic switch in all of the 6 CAF populations, when comparing the healthy mammary fat pad to the breast cancer tissue. The healthy tissue had a strong percentage of ~ 90% PDGFRα+ cells that drastically decreased as the tumour grew (Fig. 2g). Oppositely, αSMA, PDGFRβ, PDPN, FAPα and CD26+ CAF populations were all significantly appearing/expanding as soon as a tumour was established (Fig. 2d-i and Suppl. Fig. 4). Importantly, these alterations were independent on tumour type as they were observed in both 4T1 and 4T07 tumours. The second observation was that the percentage of each individual CAF marker was not stable over time. For instance, the αSMA, PDPN and PDGFRβ+ populations showed a decrease over time in the CAF+ population, with linear fitting of the data indicating similar temporal dynamics of all three markers in both 4T1 and 4T07 tumours (Fig. 2). In the case of the other three markers, FAPα, CD26 and PDGFRα, these showed divergent dynamics across time in the two tumour types. This was revealed by the 2-way ANOVA analysis demonstrating a significant interaction between the two independent variables (time and tumour type) as illustrated by the linear fitting showing the two fitted lines crossing each other (Fig. 2g-i). Interestingly, CD26 appeared to have completely opposite dynamics in the two tumour types. In 4T07 tumours, there was a significant decrease over time from D7 to D14/D21 (Fig. 2i). In 4T1 tumours, comparisons between each time points found no significant differences, yet linear fitting suggested an increase over time (slope significantly non-zero), culminating in a significant difference between tumour types at D21 (4T1 > 4T07). Similar dynamics were seen when CAF markers are examined within the Lin- population (Suppl. Fig. 5).

Taken together, data from the 128 tumours demonstrate that the development of a malignant breast tumour dramatically shifts the population of fibroblasts/CAFs in the mammary fat pad, and that once the tumour is established the dynamics of each individual CAF marker keep changing over time. PDPN, PDGFRβ, and αSMA behave similarly and all decrease over time in both tumour types, while the newest CAF marker, CD26, stands out as the only marker with intriguing opposite dynamics over time when comparing the two different tumours: increasing in the aggressive 4T1 while decreasing in 4T07. The common denominator in these analyses is that the biggest changes seem to take place between D7 to D14.

### A few CAF subpopulations dominate the TME

**Fig. 3:**
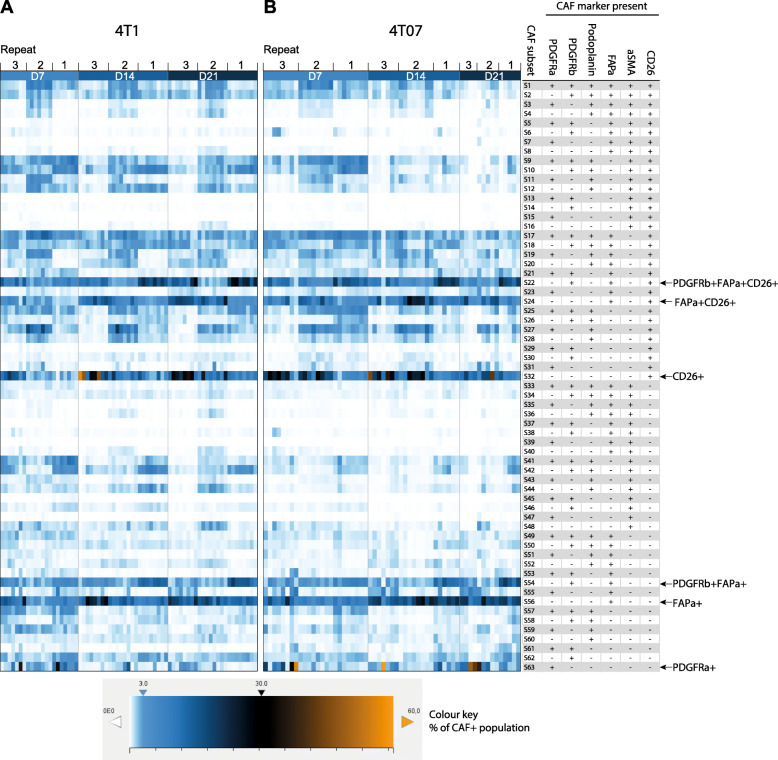
A few CAF subsets dominate the TME. **a-b** heat map visualisation of the prevalence of each theoretical Boolean CAF subset across time and tumour type within the CAF+ population. Each lane corresponds to one tumour and three independent repeats are combined as denoted above the heat maps (128 tumours in total). Each square shows the percent this CAF subset constitutes out of the CAF+ population, see colour key for reference. The heat maps clearly show that far from all the theoretical subsets are present and that a few CAF subsets dominate the TME across time and tumour type. 4T1 D7 *n* = 21, 4T1 D14 *n* = 24, 4T1 D21 *n* = 24, 4T07 D7 *n* = 24, 4T07 *n* = 21, 4T07 D21 *n* = 14

With the presence of 6 CAF markers recorded for each cell in the CAF+ population, the number of theoretically possible CAF subpopulations is 63 when using Boolean criteria. To investigate which of these theoretical subpopulations actually exist in the breast tumours, we plotted the CAF+ population of each tumour as a heat map, with each row representing one of the theoretical CAF subpopulations and the colouring representing the percentage of such subset in the tumour (Fig. 3). It is clear from the heat map that many of the 63 theoretical Boolean CAF subpopulations were actually present in both tumour types and that a few CAF subpopulations were more prevalent than others. It is noteworthy that many of the subpopulations are the same in both tumour types and remain prominent across time (Fig. 3a and b). The three independent repeats in some cases seemed to be different, suggesting a degree of systemic technical noise from one repeat to the next (batch effect), likely due to a combination of differences in antibody staining intensities, as well as instrumental performance from day to day. We assessed this and found only a very minor batch effect within the combined dataset (see Suppl. Fig. **6** and its legend).

### CAF subpopulations expressing FAPα and/or CD26 are the most prevalent

**Fig. 4:**
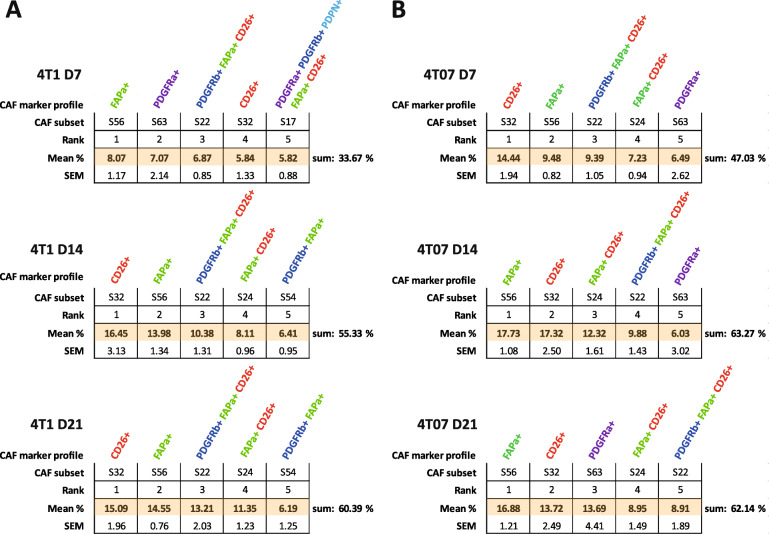
FAPα+ and CD26+ CAF subpopulations dominate the TME at all times. Top 5 most abundant Boolean CAF subpopulations (subsets) in **a** 4T1 and **b** 4T07 tumours at a given time point (within the CAF+ population). The CAF population becomes less heterogeneous over time, and FAPα (green) and CD26 (red) expressing subsets dominate the TME. Mean % and SEM from three independent repeats combined. One hundred twenty-eight tumours in total. 4T1 D7 *n* = 21, 4T1 D14 *n* = 24, 4T1 D21 *n* = 24, 4T07 D7 *n* = 24, 4T07 *n* = 21, 4T07 D21 *n* = 14. See supplementary materials for complete abundance ranking of all 63 CAF subsets

**Fig. 5:**
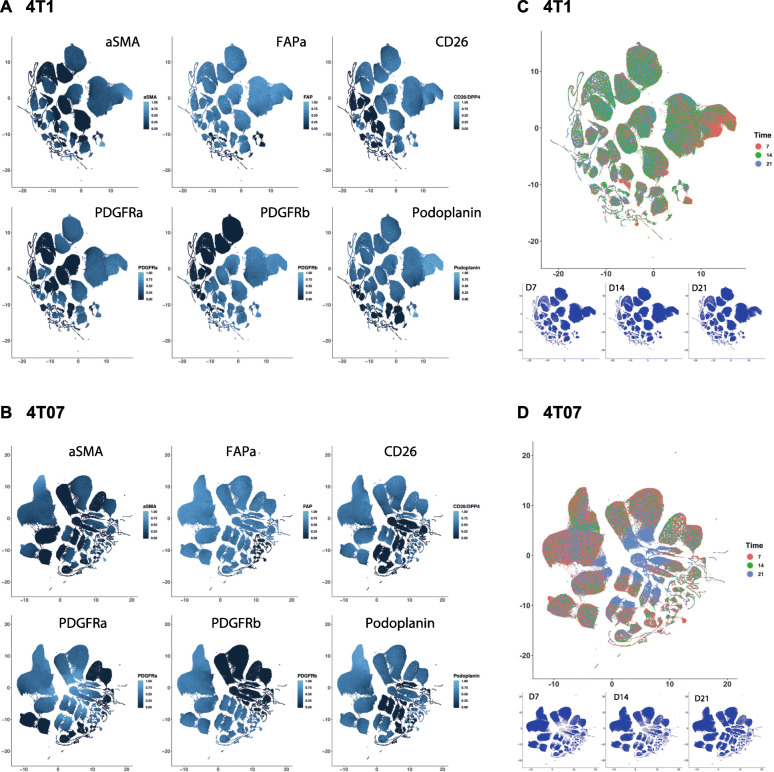
The complex CAF compartment changes as tumours grow. **a-d** Dimension reduction by UMAP of all 6 CAF markers within the Lin- population. The intensity levels were normalised into a range from 0 = no signal to 1 = strongest signal. **a** and **b** UMAP plots of each of the 6 CAF markers. **c** and **d** UMAP plots of cells from day 7, 14, and 21 separately (small plots) and combined (large plot), showing how some clusters are more abundant at a given time point. Data in A-D are from one biological repeat (rep 3), 4T1 *n* = 23 and 4T07 *n* = 19

A commonality of the most abundant CAF subpopulations across both time and tumour type was the expression of either FAPα or CD26 as seen in Fig. 4. The CAF+ population was more heterogeneous in 4T1 than in 4T07 tumours at D7. However, as the TME in both tumour types matured with time it appears that the CAF+ population becomes slightly more homogeneous over time in both 4T1 and 4T07, with the top 5 CAF subpopulations increasing in abundance to finally cover more than 60%. Including the top 10 CAF subpopulations only added 10–20% more to each time point (Table S8), thus demonstrating how relative rare most of the theoretical subpopulations are. In addition to using Boolean criteria to define CAF subpopulations, we analysed the Lin- population using UMAP dimension reduction (Fig. 5). This type of analysis does not rely on manual gating of the CAF markers into binary +/− populations, instead the intensity values of each of the 6 markers are used to guide the reduction in dimensions. Consistently, we saw only a few large clusters (subpopulations) and many smaller ones in both tumour types (Fig. 5), in agreement with the Boolean analysis in Fig. 3. This analysis also confirmed that the largest clusters expressed a high level of FAPα and/or CD26 (Fig. 5a & b).

In summary, a minority of the 63 theoretical subpopulations are present at great numbers in both tumours, and many of these are shared, and express FAPα and/or CD26, hinting at the presence of a common core of CAFs between the two breast tumour types regardless of the metastatic potential of the tumour. 4T1 tumours appear to initially have a more heterogeneous CAF+ population than 4T07 tumours, yet over time, the CAF+ population in both tumours becomes less heterogeneous. Although the data clearly indicates that a handful of subpopulations is prevailing in the TME, it is important not to forget that many of the 63 theoretical combinations are actually present in the TME, albeit in low numbers, and that 20–40% of the Lin- population is not detected by our markers (Fig. 2b).

### CAF+ population composition changes dramatically over time

**Fig. 6:**
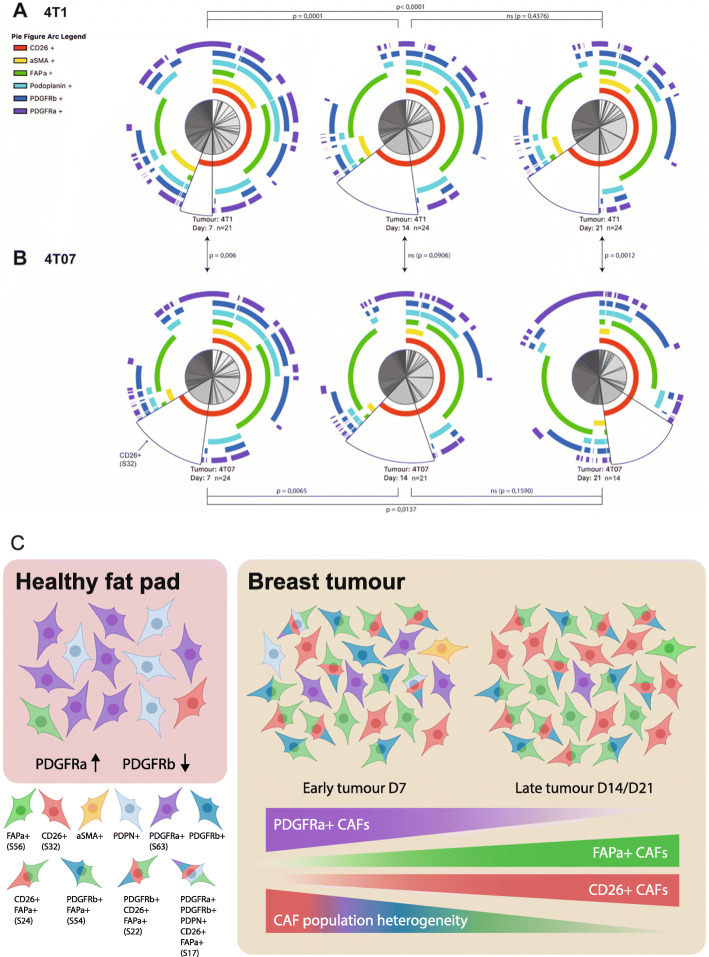
The fibroblast compartment changes dramatically from a healthy fat pad to a breast tumour. **a-b** SPICE visualisation of Boolean CAF subsets (within the CAF+ population). **a** 4T1 tumours and **b** 4T07 tumours. The arcs around the pie charts corresponds to each of the 6 CAF markers. In this way, each pie slice corresponds to one theoretical Boolean subset of CAFs, subset S32 is marked on each pie with a black border as an example. It is evident that not all of the theoretical subsets are present in the tumours, and that the overall composition of CAF subsets change as tumours matures. Furthermore, the initial difference between tumour types seen at D7 is translated into a difference at the latest stage at D21. Statistical *p*-values are from permutation testing comparing the CAF subset composition within each pie to that of another pie. Data in A-B are from three independent repeats combined (128 tumours in total), 4T1 D7 *n* = 21, 4T1 D14 *n* = 24, 4T1 D21 *n* = 24, 4T07 D7 *n* = 24, 4T07 *n* = 21, 4T07 D21 *n* = 14. **c** Model. In normal mammary fat pads fibroblasts expressing PDGFRα are the most abundant, while PDGFRβ+ fibroblasts are very scarce. Once a tumour forms this picture is reversed. As the tumour progresses the heterogeneity decreases and CAFs expressing combinations of CD26, FAPα and PDGFRβ become the dominant subpopulations

The UMAP plots revealed that there was a change in abundance of some clusters over time (Fig. 5c and d), thus we next wanted to see how CAF subpopulations co-existed in each tumour type at the different time points. To get a graphical overview of the complex composition of all 63 possible Boolean CAF subpopulations, we took advantage of the freeware SPICE (https://niaid.github.io/spice/), developed by the Roederer laboratory, to visualise our poly-chromatic FCM dataset [25]. The pie charts generated by SPICE are presented in Fig. 6a-b, with each of the possible CAF subpopulations being represented as a slice in the chart. From the first glance, it is evident that the composition of subpopulations making up the CAF+ population changes from D7 to D14 in both tumour types, and that the tumour types also appear to be different from one another. Using a permutation test to compare the pie charts it was possible to determine if the compositions of CAF subpopulation are statistically different from one another. As the dynamics of some of the single CAF markers (Fig. 3) and the UMAP plots suggested, a clear change in the relative abundances of the CAF subpopulations was taking place from D7 to D14 in both tumour types, while on the broad scale the total composition did not change significantly from D14 to D21 (Fig. 5 and 6). When comparing the two tumour types the total CAF composition in 4T1 tumours was significantly different from that of 4T07 tumours at D7. The CAF composition then became more similar at D14, before diverging significantly again at D21. What the permutation test and the pie charts did not reveal, was which of the CAF subpopulations specifically differed across time and/or tumour type, and we next moved on to look into this.

To identify the CAF subpopulations that change significantly in size across time we performed a 2-way ANOVA on the 63 theoretical Boolean subpopulations, followed by Tukey’s multiple comparisons post-test. All the subpopulations found to change significantly with time are listed in Table 4. While both 4T1 and 4T07 tumours changed from D7 to D14 (Fig. 5 and 6), 12 subpopulations were changing in 4T1 tumours compared to only 7 in 4T07 tumours (Table 4). However, many of the shifting subpopulations (S9, S17, S24, S32, and S56) were the same in both tumour types, indicating that both tumours mature their CAF+ composition in a similar way. Comparing across tumour types at each time point it became clear that the tumours were significantly different regarding the overall composition of CAF subpopulations (see pie charts in Fig. 6a-b), but that this compositional difference was mostly made up by tiny subpopulations (Table 5). However, there were two noteworthy substantial differences between 4T1 and 4T07 tumours; at D7, CD26 single positive CAFs (S32) were almost 2.5 times as abundant in 4T07 compared to 4T1 tumours (14.4% vs 5.8%), and at D21 the PDGFRα single positive CAFs (S63) subpopulation was more than 9 times as abundant in 4T07 compared to 4T1 tumours (13.7% vs 1.5%) (Table 5).

**Table 4:**
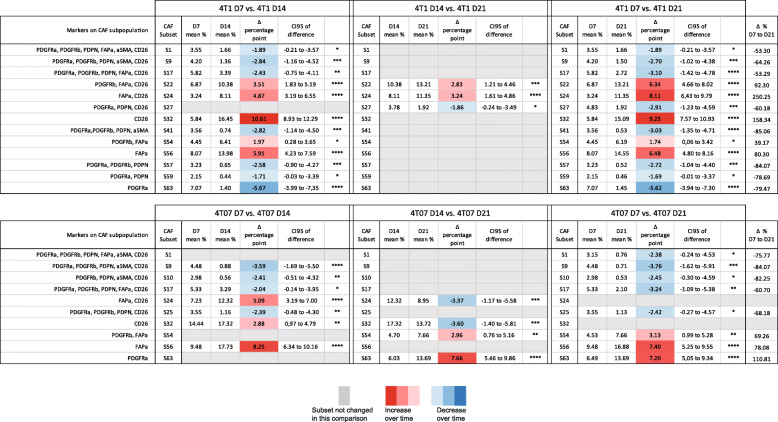
CAF subsets that change over time within each tumour type. All CAF subpopulations that were found to change significantly by 2-way ANOVA and Tukey’s multiple comparisons post-test are listed below. The mean difference in percentage point is colour-coded with red indicating an increase from the earlier to the later time point, and blue indicating a likewise decrease over time. Grey fields indicate that the subpopulations did not change over time in that particular comparison. Δ % = change in percent from D7 to D21. 128 tumours in total, 4T1 D7 *n* = 21, 4T1 D14 *n* = 24, 4T1 D21 *n* = 24, 4T07 D7 *n* = 24, 4T07 *n* = 21, 4T07 D21 *n* = 14

**Table 5:**
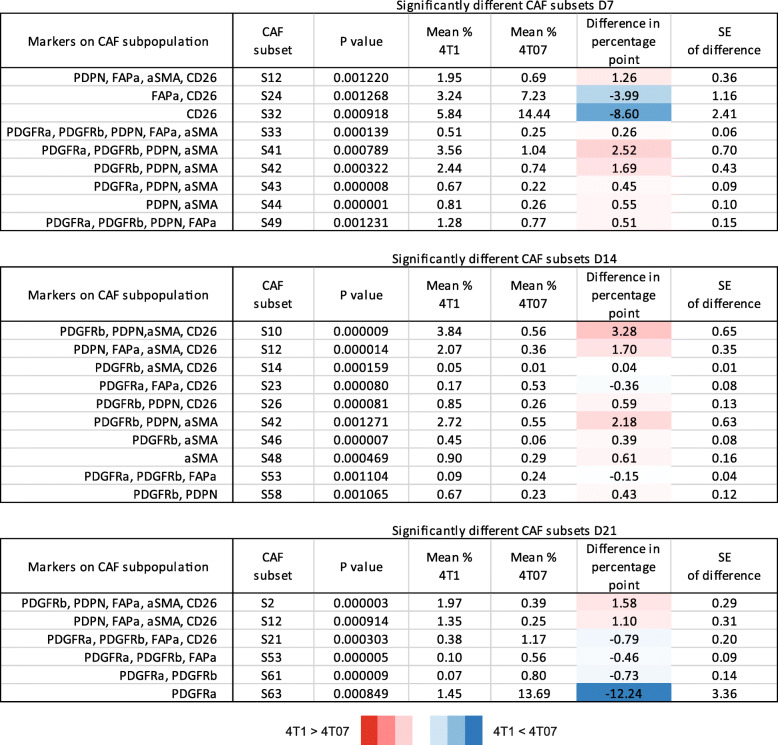
All CAF subsets that differ in size between 4T1 and 4T07 tumours at day 7, 14 or 21. CAF subpopulations that differ between the tumour types were identified by multiple t-tests without assuming equal variance and using the FDR approach (Q = 1%) to correct for multiple comparisons. The mean difference in percentage point is colour-coded with red indicating the CAF subpopulation to be larger in 4T1 than in 4T07, and vice versa with blue. One hundred twenty-eight tumours in total, 4T1 D7 *n* = 21, 4T1 D14 *n* = 24, 4T1 D21 *n* = 24, 4T07 D7 *n* = 24, 4T07 *n* = 21, 4T07 D21 *n* = 14

In summary, we find that the CAF population identified by protein expression is co-evolving along with the TME (see model in Fig. 6c). Analysis of the 128 tumours revealed temporal changes in abundance of many CAF subpopulations in both 4T1 and 4T07 tumours, and several of these were common to both tumour types. Two CAF subpopulations were markedly different in abundance when comparing tumour types (S32 and S63), supporting the hypothesis that cancer cell intrinsic factors affect the relative size of some CAF subpopulations.

## Discussion

In this study, we developed a multicolour FCM workflow allowing us to dissect the heterogeneity of CAFs based on protein expression, and utilised this to show how 6 CAF markers can be used to detect numerous CAF populations within murine TNBC tumours. We provide evidence for the temporal co-evolution of the CAF population as the tumours mature, observing that the expression of the 6 CAF markers all change over time, and the dynamics for each marker differs depending on tumour type and time (see Fig. 6c for key findings). Of note, we see a striking shift from a prevalence of PDGFRα+ fibroblasts in healthy mammary fat pads, to PDGFRβ+ CAFs in tumour tissue. Moreover, we also see examples of tumour type affecting the relative size of some of the subpopulations making up the total CAF pool, supporting the hypothesis of cancer cell intrinsic factors being important for shaping the CAF compartment. Lastly, we identify CD26 as a novel and abundant CAF marker only matched by FAPα as the most prevalent CAF marker in our murine models of TNBC. By employing a negative selection strategy and focusing on surface markers our approach is readily applicable to other mouse models of solid cancer, and it provides an excellent starting point for functional studies of CAF subpopulations, as well as for the continued search for novel CAF markers. Importantly, the implementation of our 6 CAF markers also identifies all CAF populations described in a murine melanoma study, but also recognizes temporal CAF alterations taking place during the development of melanomas (Suppl. Fig. 7) [34].

### The vast heterogeneity of CAFs

Since the initial report on different subpopulations of αSMA+ myofibroblasts in breast cancer [35], it is now becoming clear that CAFs come with different properties and that a heterogeneous population exists within a single breast tumour [8–15, 17, 18, 36]. Here, we have presented data showing the existence of numerous CAF subpopulations based on protein expression in murine models of TNBC, expanding on previous findings within breast cancer, which have proposed the existence of only 2 to 4 major CAF populations [8, 10, 18].

We chose a negative selection approach in order to acquire as diverse a population of fibroblasts/CAFs as possible, and found the Lin- population to be stable over time in tumours (Fig. 2). It is important to remember that while the percentages of Lin- cells may remain stable over time, the overall cellularity increases as the tumour grows, primarily due to rapid proliferating cancer cells, and thus the total number of Lin- is increasing drastically. This fact explains why healthy fat pads have a higher percentage of Lin- cells than tumours (10% in healthy FP, 5.5% in 4T1, and 8.7% in 4T07) (Fig. 2 & Suppl. Fig. 2), since the fibroblasts are not being ‘diluted’ by cancer cells. The relatively high numbers of Lin- cells in our study clearly emphasise the heterogeneity of the CAF population, and can be explained by our exclusion of CAFs expressing S100A4, Thy-1, Cav-1 and other markers in our study.

Historically, αSMA expression has become almost synonymous with the term CAF and until recently, many studies only looked at αSMA expression to define CAFs, and extrapolated their findings to all CAFs. It therefore came as a surprise that the percentage of αSMA+ CAFs in our tumour models was lower than expected. However, these findings are supported by scRNA-seq of 4T1 tumours showing two distinct CAF populations with no expression of αSMA [16]. In addition, several other scRNA-seq studies have also identified CAF populations with no or very low expression of αSMA [8, 10, 13, 18, 37]. As a matter of fact, the newly described inflammatory CAFs (iCAFs) population is characterised by no or low levels of αSMA [37, 38]. Moreover, the CAF-S2 and CAF-S3 populations found in human luminal A breast cancer show no/low expression of αSMA, as compared to the more abundant expression in HER2+ and TNBC [10, 14]. It is therefore clear that single-colour staining of αSMA is no longer sufficient to describe CAFs per se, rather the αSMA-staining is limited to describe the myofibroblastic (myCAF) subpopulation.

### A switch in PDGFR subtype between normal and tumour tissue

PDGFRα has been reported as a marker of resident mammary fibroblasts [8, 36, 39], as a general fibroblast marker [40], and as a CAF marker [38, 41]. Our study clearly supports PDGFRα as a marker of resident mammary fibroblasts as we see about 90% PDGFRα+ cells in the CAF+ population in normal fat pads, while only around 5–15% are PDGFRβ+ cells (Fig. 2 and Suppl. Fig. 5). On the other hand, as the tumour develops, the tumour becomes highly enriched in PDGFRβ+ CAFs, while dramatically decreasing the numbers of PDGFRα+ CAFs (Fig. 2). This is consistent with other studies showing a relative loss of PDGFRα+ CAFs during breast cancer progression in mice [8, 36]. Our findings support the notion that PDGFRβ may primarily be a marker of CAFs while PDGFRα may distinguish more resting fibroblasts [30, 39, 42]. Our murine data are also in agreement with findings in human breast cancer, showing reduced levels of PDGFRα and the concomitant increase in PDGFRβ expression as the disease progresses, which also acts as a strong marker of high-risk ductal carcinoma in situ (DCIS) [43]. In another clinical study, high expression of PDGFRβ correlated with oestrogen receptor (ER) negativity and increased risk of recurrence [44]. Thus, these findings suggest that our two murine TNBC progression models mirror clinically relevant findings.

### CD26+ CAFs are abundant new members of the CAF family

The two closely related cell surface glycoprotein receptors, FAPα and CD26 are both involved in proteolytic remodelling of the ECM and increased invasiveness of cells. Importantly, both receptors can form heterodimers creating complexes with both FAPα and CD26 catalytic activity [32]. In both our TNBC models, we observe increased numbers of CAFs expressing either or both of these two cell surface proteases. Indeed, 40–60% of CAFs express these receptors (Fig. 2 and 3). In human breast cancer, the only CAF population suggested to express high levels of FAPα and CD26 is the immune suppressive CAF-S1 population [10]. However, the complexity of FAPα-positive CAFs is emphasised by the identification of 8 clusters of the FAPα-positive CAF-S1 population exhibiting distinct signatures, which differentially accumulated in different breast cancer subtypes [13]. Adding to this complexity, it was recently shown that in 4T1 tumours, FAPα+PDPN+ CAFs are capable of producing nitric oxide (NO), controlling the proliferation of effector T-cells, thus helping to create an immunosuppressive TME in TNBC [11]. Additionally, CD8+ T-cells were in close contact with PDPN+ CAFs in the collagen-rich capsule of 4T1 tumours, and thus sequestered from the cancer cells in the centre of the tumour and unable to be effective. However, in both our models we observe increased numbers of FAPα+ CAFs but significant less PDPN+ CAFs as the tumours developed, underlining the difficulty in dissecting the biological function of FAPα+PDPN+ CAFs in breast cancer.

CD26 on the other hand is a marker of interlobular fibroblasts in human mammary tissue [33]. Indeed, we observe a clear population of CD26+ fibroblasts in healthy fat pad that significantly increases with tumour progression. In late stage tumours (D14 and D21), 3 of the 5 most abundant CAF subpopulations in both 4T1 and 4T07 tumours are CD26+ (Fig. 4), and we observe a trend towards an increase over time of CD26+ CAFs in 4T1 tumours, and a decrease over time of the same population in the less aggressive 4T07 tumours (Fig. 2), warranting further studies into the functional phenotype of these CD26+ CAF subpopulations. Indeed, Costa et al. recently demonstrated that CD26 is an important cell surface protein in human breast cancer, promoting the differentiation and activation of CD25 + FoxP3 T-cells, and thereby directly inhibiting the proliferation of effector T-cells [10]. Other studies have shown that active CD26 cleaves the chemokine CXCL10 turning it into an antagonist for its cognate CXCR3 receptor, effectively disrupting the chemotaxis that otherwise recruits CXCR3+ effector T-cells to the tumours [45]. These data emphasise the importance of CD26 as an important marker and important regulator of immunosuppressive CAFs in human breast cancer. Moreover, treating melanoma or colon cancer bearing mice with the FDA approved CD26 inhibitor Sitagliptin had a synergistic effect together with immunotherapy, due to improved cytotoxic T-cell recruitment to the tumours [45]. In a subcutaneous EMT6 model of breast cancer, CD26 inhibition also slowed tumour growth, but here no increase in T-cell influx was detected, rather an increased recruitment of eosinophils to the tumours was essential for Sitagliptin mediated control of tumour growth [46]. Interestingly, diabetic patients taking CD26 inhibitors to control their blood glucose levels, have a lower risk of developing breast cancer [47], and patients already diagnosed with breast cancer have a longer metastasis-free survival if they are using CD26 inhibitors [48]. These findings emphasise that further analysis of the role of CD26 in immune modulation in breast cancer and tentatively suggest that the aggressive 4T1 tumours may build up a more immune suppressive environment, as compared to the less aggressive 4T07 tumours.

An alternative role of CD26+ CAFs in breast cancer could potentially be linked to the desmoplastic reaction of the tumour tissue. CD26+ fibroblasts are responsible for the fibrosis of scar formation after cutaneous wound healing [49], and evidence supporting a similar fibrotic function of CD26+ CAFs in breast cancer was recently presented in the context of capsular contracture in implant-based reconstructive surgery, demonstrating that CD26+ fibroblasts sorted from human breast capsules produce more collagen-1a1 and have a higher expression of fibrogenic genes than sorted CD26- fibroblasts [50]. In future studies, it will be interesting to see whether CD26+ CAFs are indeed involved in the desmoplastic reaction. On the contrary, a recent study in human breast cancer demonstrated a considerable lower expression of CD26 in αSMA-rich tumour areas, as compared to the non-cancerous stromal regions of the breast. Despite the reduced CD26 expression observed in this study, 75 out of 193 patients still expressed moderate/significant levels of CD26 in the breast tumours [51]. Interestingly, the study suggested that TGF-β and SDF-1 signalling suppresses CD26 expression in CAFs, and it will therefore be important to characterise the biological role of CD26+ CAF populations during immune suppression and contractile/ECM remodelling.

### Temporal co-evolution of the CAF population as tumours grow

The TME changes as tumours progress, supporting the cancer cells in overcoming new challenges such as securing sufficient blood supply or keeping the immune cells at bay. CAFs have been implicated in many of these tumour-promoting processes [29, 52], yet reports studying the temporal co-evolution of CAFs as tumours progress are very limited. Our temporal analysis clearly demonstrates that CAF populations dynamically evolve during the development of cancer. Our findings are supported by the analysis of isolated PDPN+ and S100A4+ CAFs from the 4T1 model, demonstrating changes in transcriptional programs over time, transitioning from an early immunoregulatory program to late wound-healing and antigen-presentation programs [12]. A temporal analysis of CAFs in the MMTV-PyMT breast cancer model also touched upon the concept of CAF co-evolution, and found that fibroblasts isolated from progressive stages of tumour development showed increasing extracellular matrix remodelling abilities [53].

By comparing all possible Boolean CAF subpopulations, we reveal that early stage tumours differ significantly from late stage tumours in the composition of their CAF population. This was true both for 4T1 and 4T07 tumours, hinting at a substantial recruitment/education of CAFs at early stages in our two models of TNBC (Fig. 2, 5 and 6). However, only a few subpopulations differ between the two tumour types, and most of these changes occur in subpopulations of low abundance (Tables 4 and 5). The small degree of disparity between the two cell lines may not be that surprising, as they share the same ancestry [19]. Nonetheless, in early tumours the most prominent difference between 4T1 and 4T07 is observed in the CD26 single positive CAFs (5.8% vs 14.4%, respectively), while the PDGFRα single positive CAFs nearly vanish in 4T1 tumours, as compared to 4T07 tumours (1.5% vs 13.7%, respectively), see Table 5. This shows that cancer cell intrinsic factors do affect the size of CAF subpopulations, and it is interesting to speculate whether the differences are reflected by the ratio of educated resident fibroblasts vs. de novo recruited precursors cells e.g. coming from the bone marrow. Further studies will also help to figure out if the specific subpopulations that differ between 4T1 and 4T07 tumours contribute to the metastatic potential of the tumour; e.g. whether CD26+ CAFs are generating an immunosuppressive TME in TNBC as suggested previously [10]. A remaining open question is whether PDGFRα single positive CAFs in 4T07 are resident fibroblasts that have yet to become fully activated CAFs, or maybe represent a CAF population with anti-tumorigenic properties?

## Conclusion

In conclusion, we have used an FCM strategy to isolate and extensively investigate CAF heterogeneity in living tissues, allowing for functional characterisation of temporal dynamics in CAF populations in murine TNBC progression. Our study adds to the body of growing evidence against a simplistic single-marker definition of CAFs by identifying the co-existence of multiple CAF subpopulations in TNBC based on the detection of 6 CAF protein markers. While a few CAF populations dominate the TME and are common to both 4T1 and 4T07 tumours, many subtler subpopulations are nevertheless detectable and may have important functions. Our data identifies CD26 as a new interesting CAF marker in murine TNBC, and future studies should dissect the function of CD26+ CAFs and other prominent subpopulations.

## Supplementary Information

**Additional file 1: Supplementary Figure 1**. Setting of CAF marker gates using fluorescent minus one (FMO) controls. **A**) CAF marker gates shown on a fully stained tumour sample. αSMA is identified based on DsRed-Express fluorescence from the αSMA-RFP reporter mouse, and **B**) the same gates shown on an unlabelled tumour sample. **C**) The gates are set on a sample labelled with all but the one marker the gates is being set for (FMO labelled), and placed so that less than 0,03% percent of the events fall within the gate. All gating were made on live cells. For each of the three repeats FMO controls were prepared fresh and used to set the CAF marker gates, here an example from repeat 3 (LSR06) is shown.

**Additional file 2: Supplementary Figure 2**. Cell surface marker analysis of mouse breast cancer cell lines. **A**) Cell surface marker FCM analysis of 4T1, and 4T07 cell lines. The red graph shows the unstained control and blue the stained cell sample, *n* = 1. The histograms are not part of the gating strategy used for the experimental samples. **B**) Derivation of triple-negative breast cancer cell lines from a single MMTV-driven spontaneous tumour. **C**) Viability of tumour single cell suspensions across time and tumour type, all three repeats combined, Tukey style box and whisker plots with line at the median and ‘+’ at the mean. **D)** Lin- population out of live cells, **E)** CAF+ population out of Lin- cells, **F)** CAF+ out of live cells.

**Additional file 3: Supplementary Figure 3.** Validation of our CAF marker gene panel in single cell gene expression datasets. **A)** UMAP of mouse mammary gland cells (data from [27]) color-coded by major cell lineages. **B)** Violin plots showing the log-normalized expression levels of CAF marker genes in mouse mammary gland cell types. **C)** The average Z-score of ACTA2, FAP, PDGFRA, PDGFRB, CD26/DPP4 and PDPN overlaid on the single cell mouse mammary gland UMAP plot from A). **D)** Violin plot of the expression levels of the average Z-score of ACTA2, FAP, PDGFRA, PDGFRB, CD26/DPP4 and PDPN in mouse mammary gland cell types. **E)** Representation of 14 human breast cancers (data from [15]) in UMAP space coloured according to cell type. **F)** The average Z-score of ACTA2, FAP, PDGFRA, PDGFRB, CD26/DPP4 and PDPN overlaid on UMAP as in E). Green colour bar, average Z-score. **G)** Violin plot indicating the distribution of the CAF signature (ACTA2, FAP, PDGFRA, PDGFRB, CD26/DPP4 and PDPN) Z-score in breast cancer cell types. **H)** UMAP of five primary human breast cancer samples (data from [18]) coloured according to cell type. **I)** Expression level of the average Z-score of our CAF panel plotted onto the UMAP from H). Green colour bar, average Z-score. **J)** Violin plot displaying the expression of the CAF marker Z-score gene signature across all cell types annotated in this dataset.

**Additional file 4: Supplementary Figure 4**. Cell population sizes across tumour type without time dimension to see biological variance. **A)** Percentage of CAF+ cells expressing the respective CAF marker. All plots show three independent repeats combined as Tukey style box and whisker plots, with a line denoting the median and a ‘+‘denoting the mean.

**Additional file 5: Supplementary Figure 5**. CAF marker dynamics across time and tumour type within the Lin- population. A-F) Percent of cells positive for the respective CAF marker in healthy mammary fat pad (D0) and 4T1 and 4T07 tumours across the different time points. Tukey box plots of two (healthy samples) or three independent repeats (tumour samples) combined. The boundaries of the Tukey style box go from the 25th to the 75th percentile, and the median is depicted by a line. The whiskers extend from the value closest to the 25th percentile minus 1.5 times the interquartile range (IQR = difference between 25th to 75th percentile), to the value closest to the 75th percentile plus 1.5 x IQR. Any values outside this range is plotted as individual points. The healthy samples provide a D0 reference point, but all the statistics are only concerning the tumour samples. Fitting a straight line to the data, testing if one line fits both 4T1 and 4T07 tumours to indicate no difference, or if slope and/or y-intercept differ if two lines better capture the dataset. Fitted lines are shown with hashed out 95% confidence bands (CB95), with dots indicating the observed mean at the respective time point. 2-way ANOVA was run once for each marker separately to look for interaction and over-all effect of tumour type and/or day. To determine statistical significance between time points within each tumour type (intra-tumour time comparisons) a 2-way ANOVA with Tukey’s multiple comparisons post-test was run on all the CAF markers combined, once for 4T1 tumours and once for 4T07 tumours. To determine if markers differed between tumour types, multiple unpaired, two-tailed t-tests without assuming equal variance and with FDR correction (Q = 1%) was run for each of the three time points (inter-tumour comparison). * = *p* < 0.05, ** = *p* < 0.01, *** = *p* < 0.001, **** *p* < 0.0001. Healthy *n* = 12, 4T1 D7 *n* = 21, 4T1 D14 *n* = 24, 4T1 D21 *n* = 24, 4T07 D7 *n* = 24, 4T07 *n* = 21, 4T07 D21 *n* = 14.

**Additional file 6: Supplementary Figure 6.** To assess if a problematic batch effect was present in the dataset combined of the three repeats, we made a data normalization within each repeat by using the mean value of each CAF marker in the 4T1 D7 group, and then combined the resultant ratios in a new normalised dataset. We then performed a 2-way ANOVA with Tukey’s multiple comparisons post-test like the one done on the raw combined dataset in Fig. 2. If there were prominent batch effects within the combined dataset, then statistical comparisons on the ratios would yield different results than the same analyses performed on the raw percentages. Two out of 36 comparisons (5.6%) went from being significant in the raw dataset to not-significant in the normalised dataset, and five comparisons (13.9%) turned out statistically significant in the normalized dataset compared to the raw dataset. In the remaining 86% of the comparisons, nothing or only the level of statistical significance changed. In summary, only a minor batch effect was detected within the combined dataset.

**Additional file 7: Supplementary Figure 7. (A)** A CAF-only clustering analysis visualized by UMAP, after the removal of all other cell types in the mouse melanoma TME, revealed three CAF subsets. Colours indicate the three CAF subpopulations, which were annotated as previously reported [34]. **(B and C)** Dot plots of selected genes expressed in mouse melanoma CAF subtypes **(B)** and at different time points **(C).** Intensity of colour indicates the average expression of each gene in each cluster, and the size of the dot is the fraction of cells in the cluster expressing that gene.

**Additional file 8: Supplementary Table 1.** Details of 2-way ANOVA and Tukey’s multiple comparisons post-test on the Lin- population out of live cells reported in main Fig. 2, all repeats combined. **Supplementary Table 2.** Details of 2-way ANOVA and Tukey’s multiple comparisons post-test on the CAF+ population out of Lin- cells reported in main Fig. 2, all repeats combined. **Supplementary Table 3.** Details of 2-way ANOVA and Tukey’s multiple comparisons post-test on the CAF+ population out of live cells reported in main Fig. 2, all repeats combined. **Supplementary Table 4.** Multiple t-tests pertaining to main Fig. 2, all repeats combined. Percentage cells of parent population. **Supplementary Table 5.** Multiple t-test pertaining to main Fig. 2, all repeats combined. Percentage cells positive for each CAF marker within the CAF+ population. **Supplementary Table 6** Tukey’s multiple comparisons from 2-way ANOVA on % of single CAF markers within the CAF+ populations. Data from three independent repeats combined. Significant results reported in main Fig. 2. **Supplementary Table 7** Co-expression tables of two CAF markers in 4T1 and 4T07 tumours at different time points, expanding the analysis to contain the time dimension. First CAF marker = parent gate, Subset CAF marker = child gate. CI95 = 95% confidence interval. **Supplementary Table 8**. Abundance ranking of Boolean CAF subpopulations in 4T1 tumours. Three independent repeats combined, mean % of CAF+ population. Top 5 subpopulations also presented in main Fig. 4. **Supplementary Table 9**. Abundance ranking of Boolean CAF subpopulations in 4T07 tumours. Three independent repeats combined, mean % of CAF+ population. Top 5 subpopulations also presented in main Fig. 4. **Supplementary Table 10**. Comparing Boolean CAF subpopulations between tumour types. Percentages from three independent repeats combined. Multiple t-test without assuming equal variance and with FDR correction for multiple comparisons (Q = 1%). Significant results reported in main Table 4.

## Data Availability

At present, the datasets used and analysed during the current study are available from the corresponding author on reasonable request. The flow cytometry dataset will be available in FlowRepository in the near future.

## Abbreviations

ANOVA: Analysis of variance
αSMA: Alpha-smooth muscle actin
BM: Bone marrow
CAF(s): Cancer-associated fibroblast(s)
CB95: 95% confidence band
CD49f: Integrin alpha 6 / Itga6
CI95: 95% confidence interval
CTCs: Circulating tumour cells
D7: Day 7
D14: Day 14
D21: Day 21
DPP4: Di-peptidyl peptidase 4 / CD26
ECM: Extracellular Matrix
eGFP: Enhanced green fluorescent protein
EpCAM: Epithelial cell adhesion molecule
FACS: Fluorescent activated cell sorting
FAPα: Fibroblast activation protein alpha
FCM: Flow cytometry
FDR: False discovery rate
FSC: Forward scatter
FSP1: Fibroblast specific protein 1 / S100A4
Lin-: Lineage negative (devoid of lineage markers)
MMTV-PyMT: Mouse mammary tumour virus Polyoma middle T-antigen
PDGFRα: Platelet derived growth factor receptor alpha / CD140a
PDGFRβ: Platelet derived growth factor receptor beta / CD140b
PDPN: Podoplanin
PMT(s): Photomultiplier tubes(s)
RFP: Red fluorescent protein
scRNA-seq: single cell RNA sequencing
SEM: Standard error of the mean
SSC: Side scatter
TME: Tumour microenvironment
TNBC: Triple-negative breast cancer
UMAP: Uniform Manifold Approximation and Projection
