# Supplementary figures and images for: Deciphering the temporal heterogeneity of cancer-associated fibroblast subpopulations in breast cancer
Source: J Exp Clin Cancer Res. 2021 May 20;40:175. doi: 10.1186/s13046-021-01944-4 (PMC8138934; doi:10.1186/s13046-021-01944-4)

fig S1

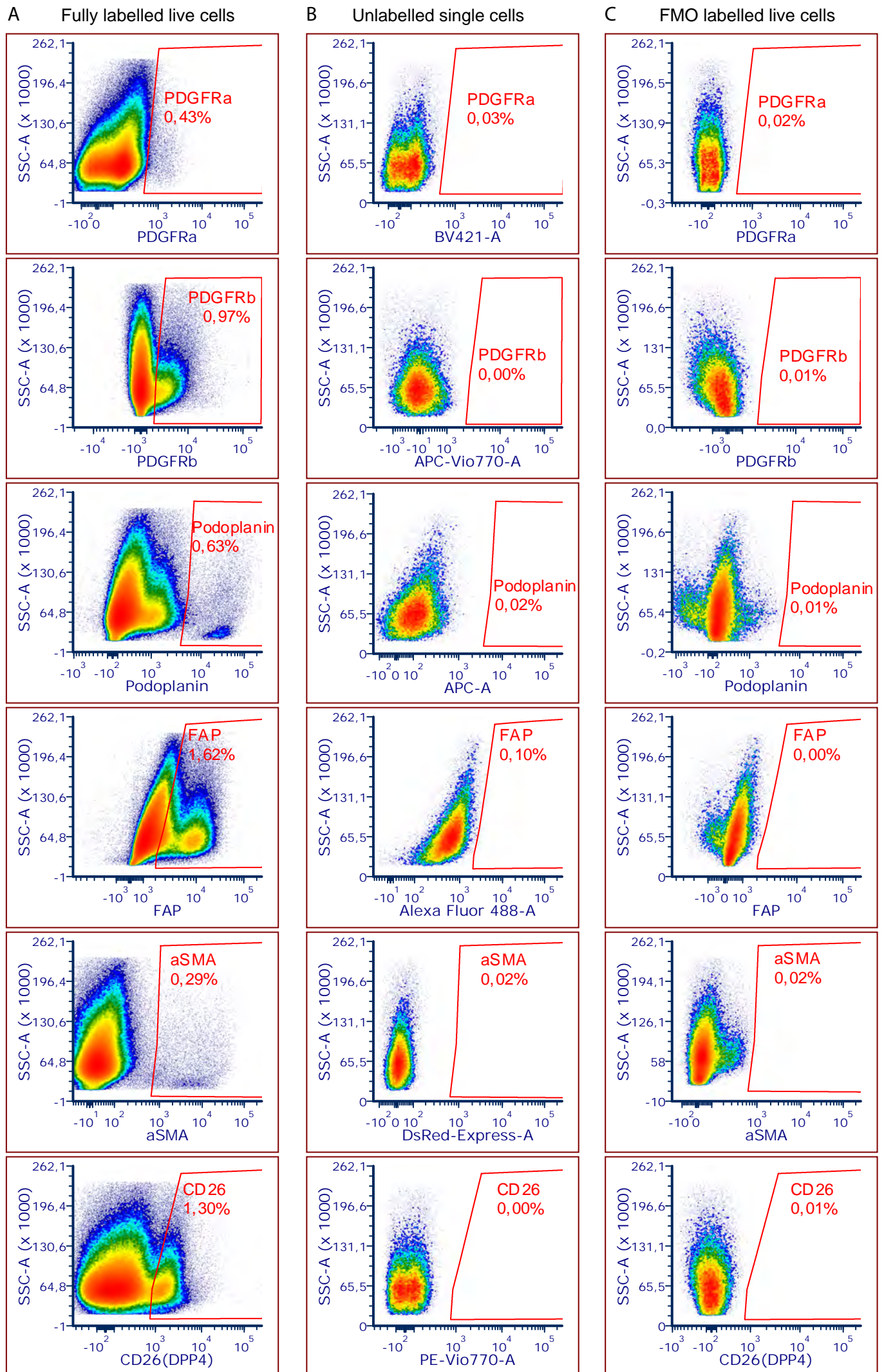

Supplement: Supplementary file 1 — Additional file 1: Supplementary Figure 1. Setting of CAF marker gates using fluorescent minus one (FMO) controls. A) CAF marker gates shown on a fully stained tumour sample. αSMA is identified based on DsRed-Express fluorescence from the αSMA-RFP reporter mouse, and B) the same gates shown on an unlabelled tumour sample. C) The gates are set on a sample labelled with all but the one marker the gates is being set for (FMO labelled), and placed so that less than 0,03% percent of the events fall within the gate. All gating were made on live cells. For each of the three repeats FMO controls were prepared fresh and used to set the CAF marker gates, here an example from repeat 3 (LSR06) is shown. [file 13046_2021_1944_MOESM1_ESM.pdf]

fig S2

**A**

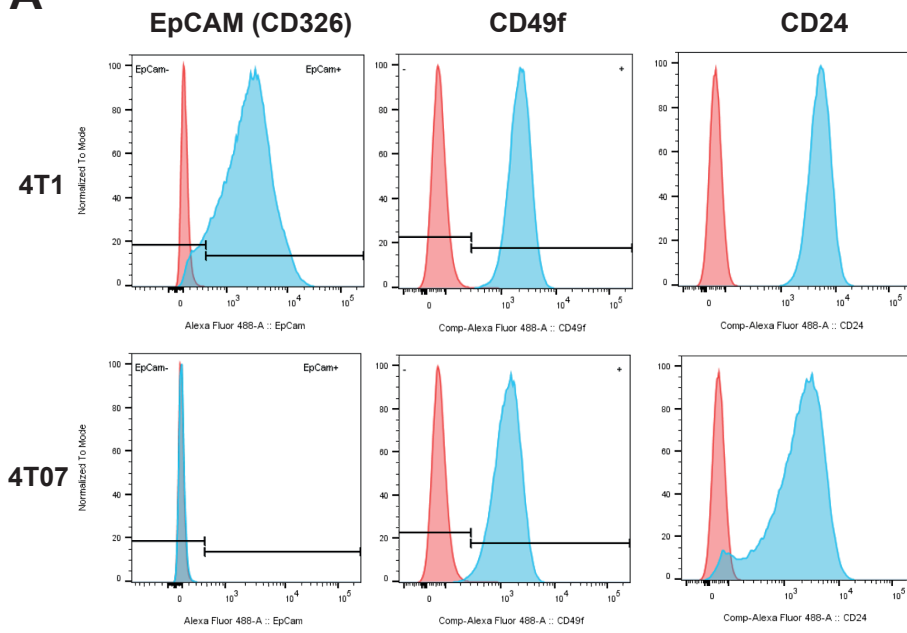

**B**

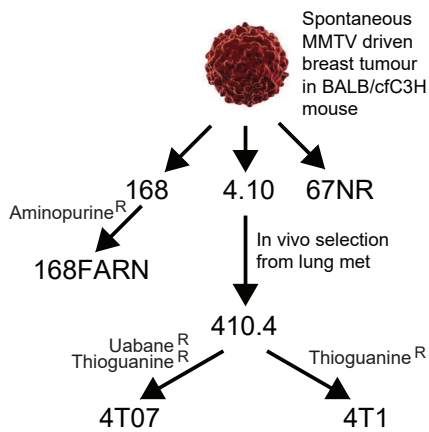

**C**

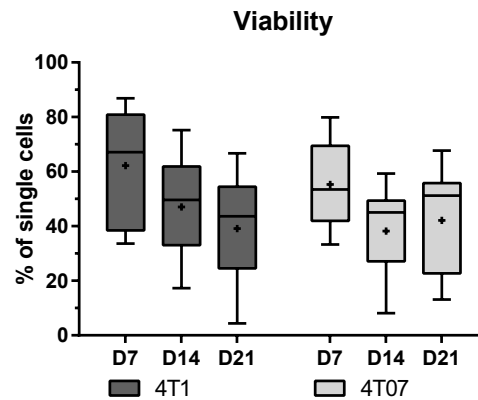

**D**

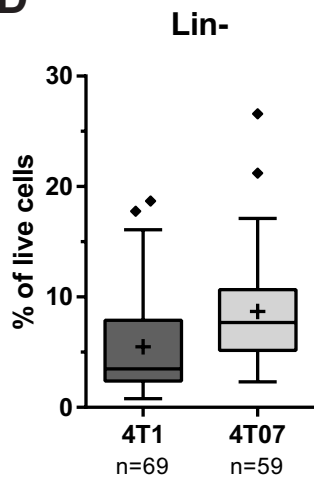

**E**

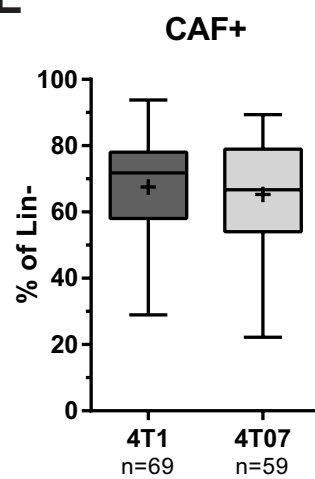

**F**

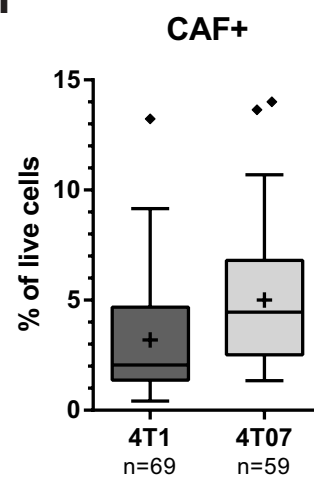

Supplement: Supplementary file 2 — Additional file 2: Supplementary Figure 2. Cell surface marker analysis of mouse breast cancer cell lines. A) Cell surface marker FCM analysis of 4T1, and 4T07 cell lines. The red graph shows the unstained control and blue the stained cell sample, n = 1. The histograms are not part of the gating strategy used for the experimental samples. B) Derivation of triple-negative breast cancer cell lines from a single MMTV-driven spontaneous tumour. C) Viability of tumour single cell suspensions across time and tumour type, all three repeats combined, Tukey style box and whisker plots with line at the median and ‘+’ at the mean. D) Lin- population out of live cells, E) CAF+ population out of Lin- cells, F) CAF+ out of live cells. [file 13046_2021_1944_MOESM2_ESM.pdf]

Fig S3

A

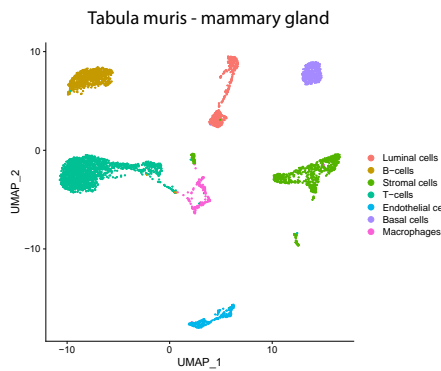

B

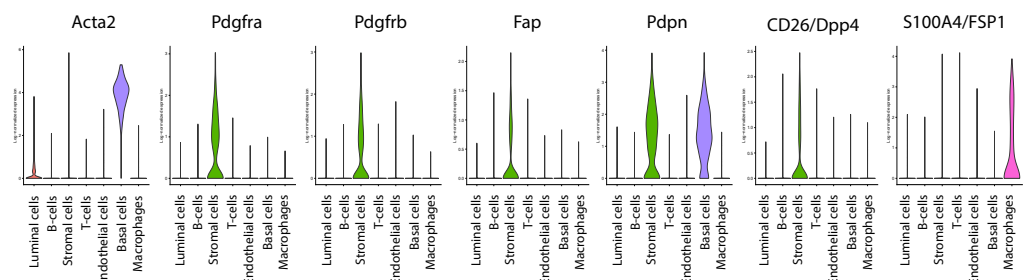

C

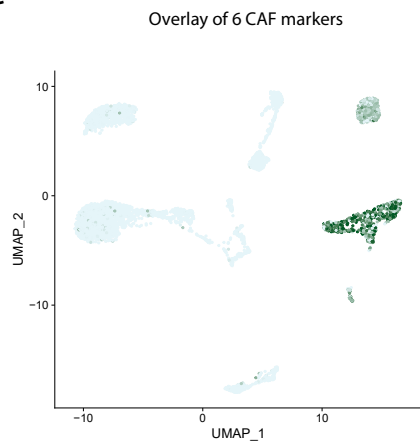

D

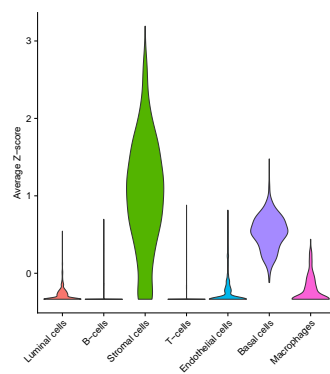

E

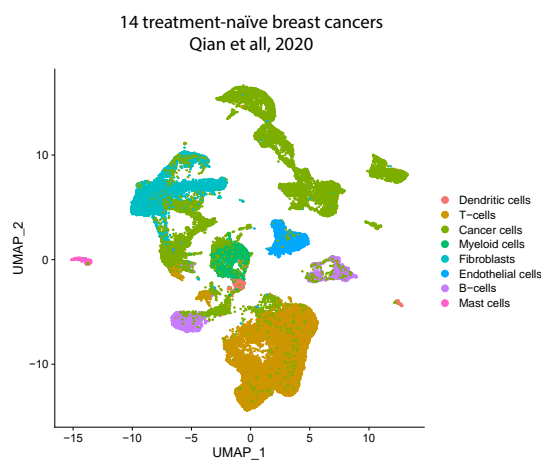

F

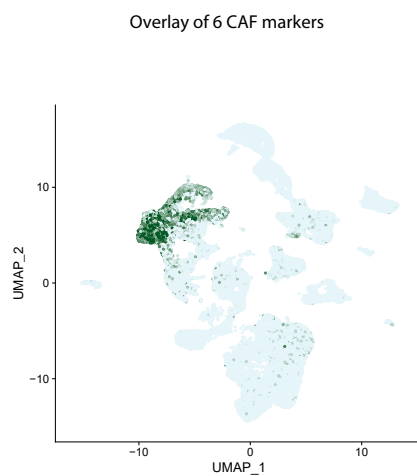

G

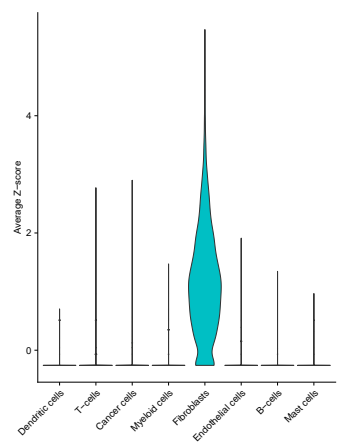

H

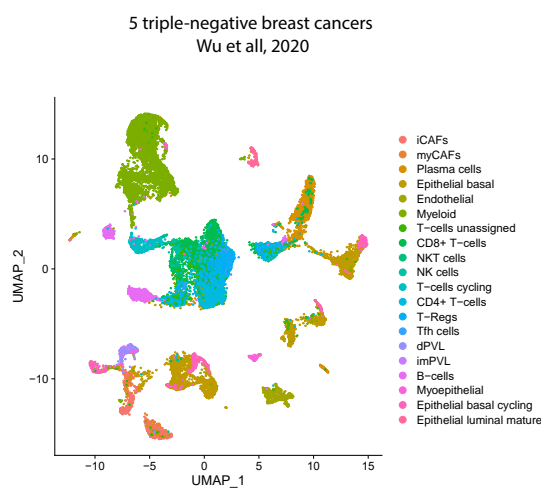

I

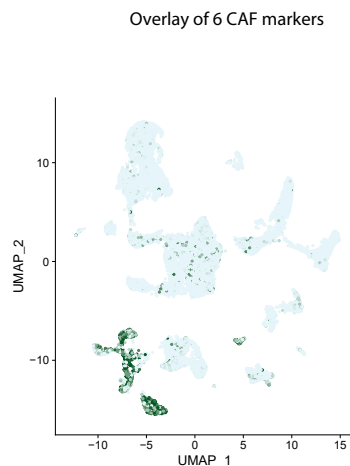

J

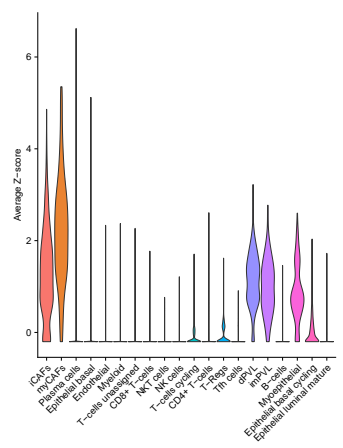

Supplement: Supplementary file 3 — Additional file 3: Supplementary Figure 3. Validation of our CAF marker gene panel in single cell gene expression datasets. A) UMAP of mouse mammary gland cells (data from [27]) color-coded by major cell lineages. B) Violin plots showing the log-normalized expression levels of CAF marker genes in mouse mammary gland cell types. C) The average Z-score of ACTA2, FAP, PDGFRA, PDGFRB, CD26/DPP4 and PDPN overlaid on the single cell mouse mammary gland UMAP plot from A). D) Violin plot of the expression levels of the average Z-score of ACTA2, FAP, PDGFRA, PDGFRB, CD26/DPP4 and PDPN in mouse mammary gland cell types. E) Representation of 14 human breast cancers (data from [15]) in UMAP space coloured according to cell type. F) The average Z-score of ACTA2, FAP, PDGFRA, PDGFRB, CD26/DPP4 and PDPN overlaid on UMAP as in E). Green colour bar, average Z-score. G) Violin plot indicating the distribution of the CAF signature (ACTA2, FAP, PDGFRA, PDGFRB, CD26/DPP4 and PDPN) Z-score in breast cancer cell types. H) UMAP of five primary human breast cancer samples (data from [18]) coloured according to cell type. I) Expression level of the average Z-score of our CAF panel plotted onto the UMAP from H). Green colour bar, average Z-score. J) Violin plot displaying the expression of the CAF marker Z-score gene signature across all cell types annotated in this dataset. [file 13046_2021_1944_MOESM3_ESM.pdf]

fig S4

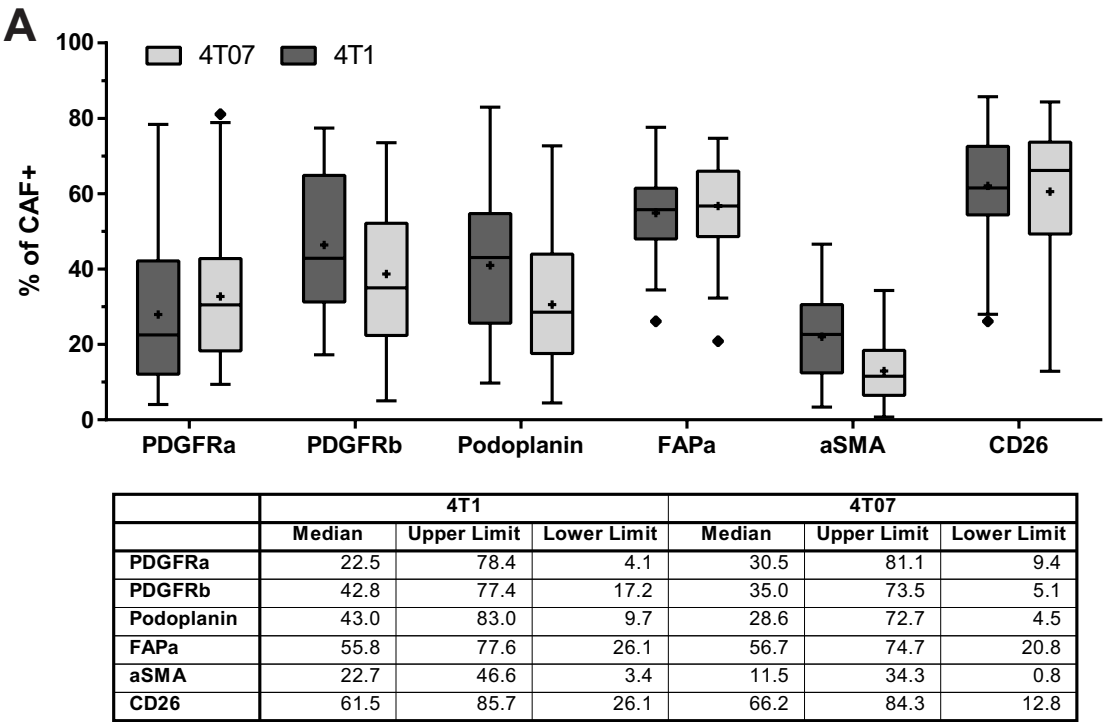

Supplement: Supplementary file 4 — Additional file 4: Supplementary Figure 4. Cell population sizes across tumour type without time dimension to see biological variance. A) Percentage of CAF+ cells expressing the respective CAF marker. All plots show three independent repeats combined as Tukey style box and whisker plots, with a line denoting the median and a ‘+‘denoting the mean. [file 13046_2021_1944_MOESM4_ESM.pdf]

fig S5

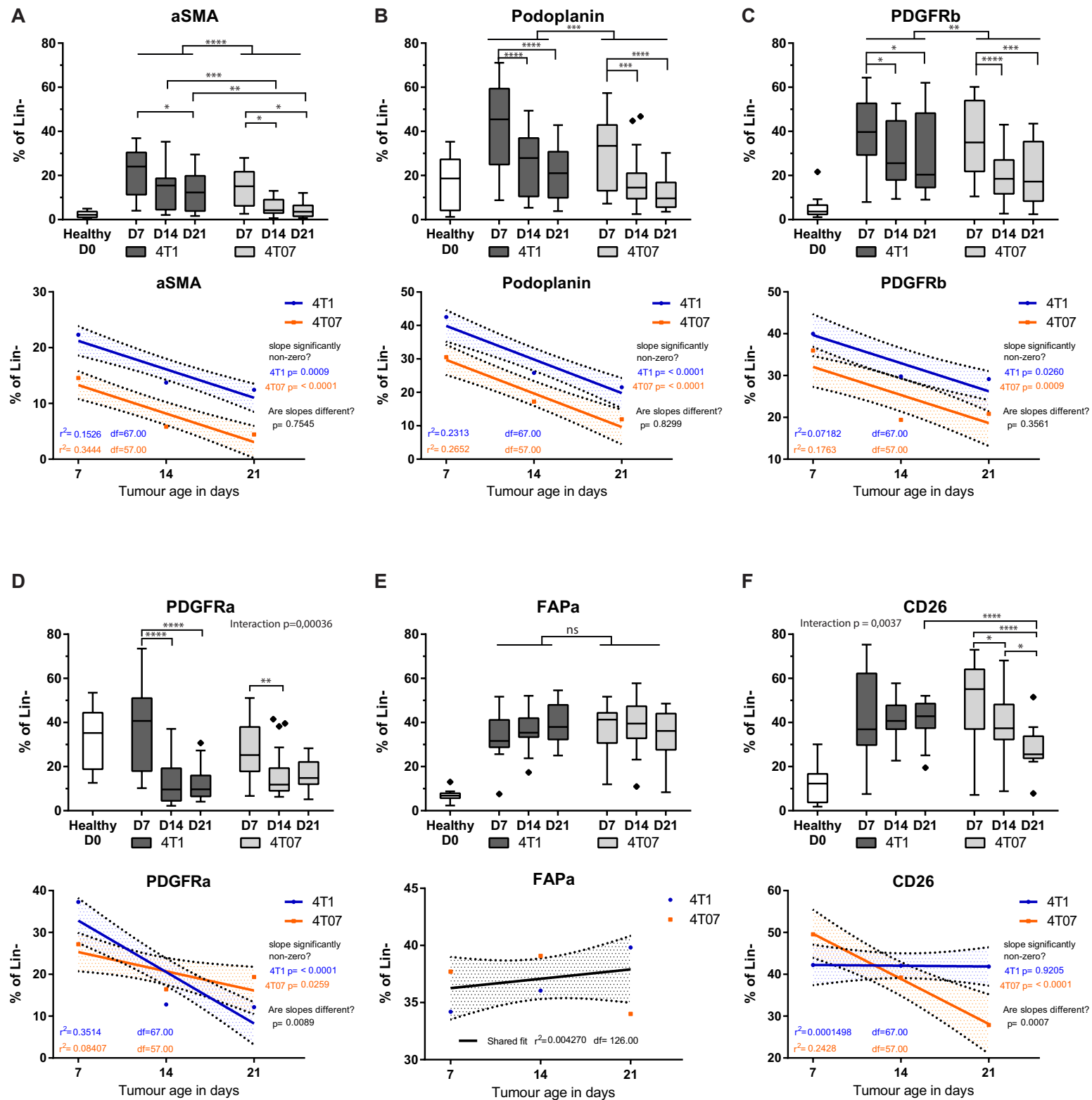

Supplement: Supplementary file 5 — Additional file 5: Supplementary Figure 5. CAF marker dynamics across time and tumour type within the Lin- population. A-F) Percent of cells positive for the respective CAF marker in healthy mammary fat pad (D0) and 4T1 and 4T07 tumours across the different time points. Tukey box plots of two (healthy samples) or three independent repeats (tumour samples) combined. The boundaries of the Tukey style box go from the 25th to the 75th percentile, and the median is depicted by a line. The whiskers extend from the value closest to the 25th percentile minus 1.5 times the interquartile range (IQR = difference between 25th to 75th percentile), to the value closest to the 75th percentile plus 1.5 x IQR. Any values outside this range is plotted as individual points. The healthy samples provide a D0 reference point, but all the statistics are only concerning the tumour samples. Fitting a straight line to the data, testing if one line fits both 4T1 and 4T07 tumours to indicate no difference, or if slope and/or y-intercept differ if two lines better capture the dataset. Fitted lines are shown with hashed out 95% confidence bands (CB95), with dots indicating the observed mean at the respective time point. 2-way ANOVA was run once for each marker separately to look for interaction and over-all effect of tumour type and/or day. To determine statistical significance between time points within each tumour type (intra-tumour time comparisons) a 2-way ANOVA with Tukey’s multiple comparisons post-test was run on all the CAF markers combined, once for 4T1 tumours and once for 4T07 tumours. To determine if markers differed between tumour types, multiple unpaired, two-tailed t-tests without assuming equal variance and with FDR correction (Q = 1%) was run for each of the three time points (inter-tumour comparison). * = p < 0.05, ** = p < 0.01, *** = p < 0.001, **** p < 0.0001. Healthy n = 12, 4T1 D7 n = 21, 4T1 D14 n = 24, 4T1 D21 n = 24, 4T07 D7 n = 24, 4T07 n = 21, 4T07 D21 n = 14. [file 13046_2021_1944_MOESM5_ESM.pdf]

Fig S7

A

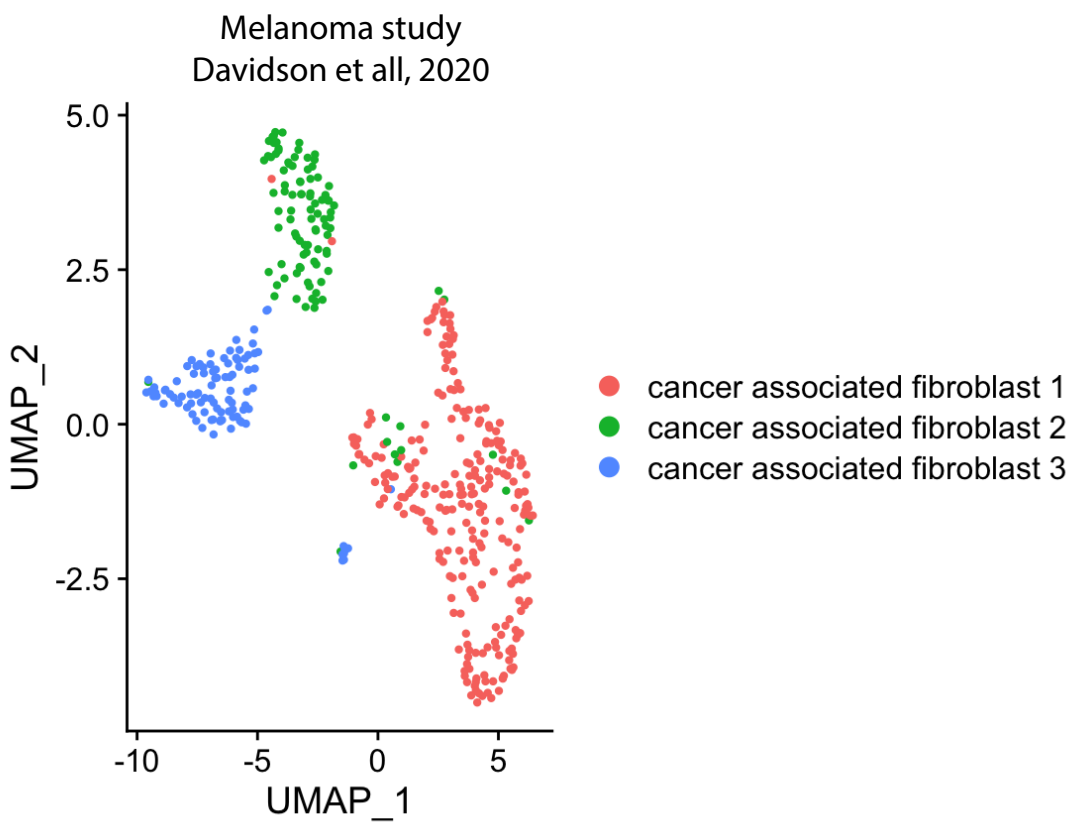

B

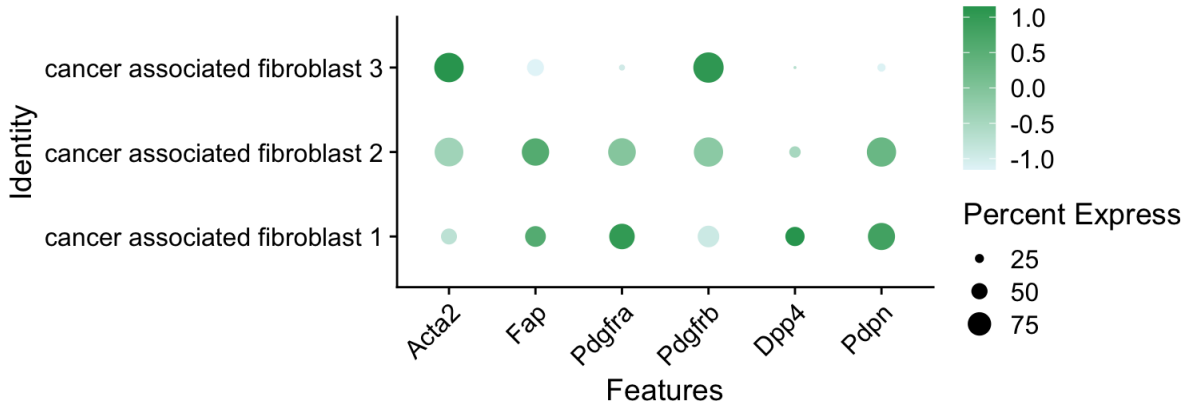

C

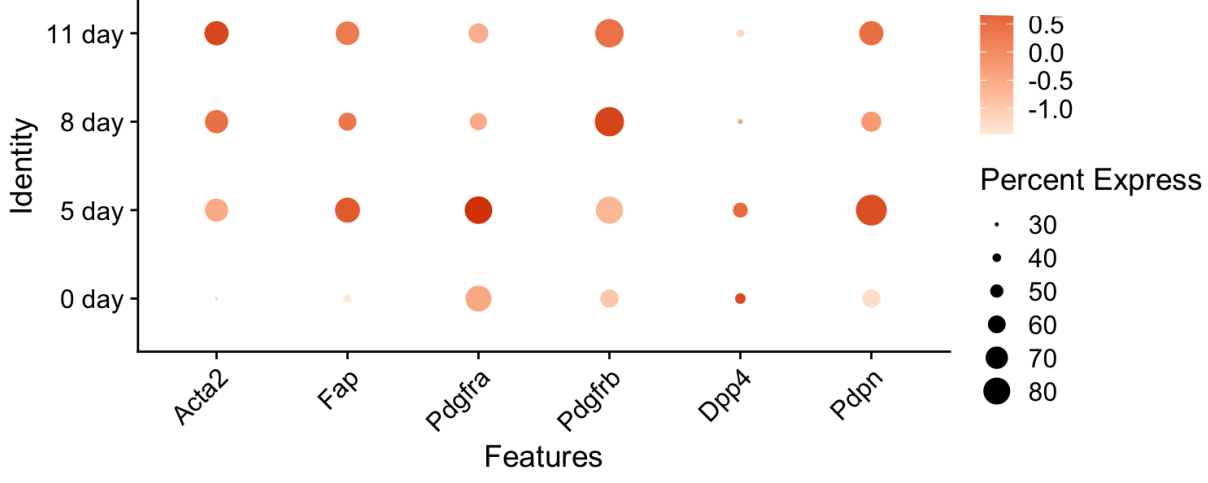

Supplement: Supplementary file 7 — Additional file 7: Supplementary Figure 7. (A) A CAF-only clustering analysis visualized by UMAP, after the removal of all other cell types in the mouse melanoma TME, revealed three CAF subsets. Colours indicate the three CAF subpopulations, which were annotated as previously reported [34]. (B and C) Dot plots of selected genes expressed in mouse melanoma CAF subtypes (B) and at different time points (C). Intensity of colour indicates the average expression of each gene in each cluster, and the size of the dot is the fraction of cells in the cluster expressing that gene. [file 13046_2021_1944_MOESM7_ESM.pdf]
