# Supplementary material for: Deciphering the temporal heterogeneity of cancer-associated fibroblast subpopulations in breast cancer
Source: J Exp Clin Cancer Res. 2021 May 20;40:175. doi: 10.1186/s13046-021-01944-4 (PMC8138934; doi:10.1186/s13046-021-01944-4)
Supplement: Supplementary file 6 — Additional file 6: Supplementary Figure 6. To assess if a problematic batch effect was present in the dataset combined of the three repeats, we made a data normalization within each repeat by using the mean value of each CAF marker in the 4T1 D7 group, and then combined the resultant ratios in a new normalised dataset. We then performed a 2-way ANOVA with Tukey’s multiple comparisons post-test like the one done on the raw combined dataset in Fig. 2. If there were prominent batch effects within the combined dataset, then statistical comparisons on the ratios would yield different results than the same analyses performed on the raw percentages. Two out of 36 comparisons (5.6%) went from being significant in the raw dataset to not-significant in the normalised dataset, and five comparisons (13.9%) turned out statistically significant in the normalized dataset compared to the raw dataset. In the remaining 86% of the comparisons, nothing or only the level of statistical significance changed. In summary, only a minor batch effect was detected within the combined dataset. [file 13046_2021_1944_MOESM6_ESM.pdf]

fig S6

Raw percentages 2-way ANOVA						Difference between raw and normalized data ?	Normalized to 4T1 D7 mean 2-way ANOVA					
Tukey's multiple comparisons test	Mean Diff. percentage point	95% CI of diff,	Signifi-cant?	*	Adjusted P Value		Tukey's multiple comparisons test	Mean Diff. norm. ratios	95% CI of diff,	Signifi-cant?	*	Adjusted P Value
**4T1**							**4T1**					
**PDGFRa**							**PDGFRa**					
4T1 Day 7 vs. 4T1 day 14	29.72	20,23 to 39,22	Yes	****	< 0,0001		4T1 Day 7 vs. 4T1 day 14	0.65	0,4848 to 0,8072	Yes	****	< 0,0001
4T1 Day 7 vs. 4T1 day 21	30.85	21,35 to 40,34	Yes	****	< 0,0001		4T1 Day 7 vs. 4T1 day 21	0.63	0,4673 to 0,7897	Yes	****	< 0,0001
4T1 day 14 vs. 4T1 day 21	1.12	-8,047 to 10,29	No	ns	0.9552		4T1 day 14 vs. 4T1 day 21	-0.02	-0,1732 to 0,1382	No	ns	0.9622
**PDGFRb**							**PDGFRb**					
4T1 Day 7 vs. 4T1 day 14	9.55	0,05991 to 19,05	Yes	*	0.0482		4T1 Day 7 vs. 4T1 day 14	0.20	0,04247 to 0,3649	Yes	**	0.0088
4T1 Day 7 vs. 4T1 day 21	10.50	1,011 to 20,00	Yes	*	0.0259		4T1 Day 7 vs. 4T1 day 21	0.23	0,06594 to 0,3883	Yes	**	0.0029
4T1 day 14 vs. 4T1 day 21	0.95	-8,219 to 10,12	No	ns	0.9677		4T1 day 14 vs. 4T1 day 21	0.02	-0,1323 to 0,1792	No	ns	0.9331
**Podoplanin**							**Podoplanin**					
4T1 Day 7 vs. 4T1 day 14	17.27	7,772 to 26,76	Yes	****	< 0,0001		4T1 Day 7 vs. 4T1 day 14	0.34	0,1831 to 0,5055	Yes	****	< 0,0001
4T1 Day 7 vs. 4T1 day 21	23.78	14,29 to 33,27	Yes	****	< 0,0001		4T1 Day 7 vs. 4T1 day 21	0.44	0,2836 to 0,6059	Yes	****	< 0,0001
4T1 day 14 vs. 4T1 day 21	6.52	-2,656 to 15,69	No	ns	0.2175		4T1 day 14 vs. 4T1 day 21	0.10	-0,05530 to 0,2562	No	ns	0.2839
**FAPa**							**FAPa**					
4T1 Day 7 vs. 4T1 day 14	-8.43	-17,92 to 1,067	No	ns	0.0936	yes	4T1 Day 7 vs. 4T1 day 14	-0.21	-0,3662 to -0,04386	Yes	**	0.0082
4T1 Day 7 vs. 4T1 day 21	-15.40	-24,89 to -5,905	Yes	***	0.0005		4T1 Day 7 vs. 4T1 day 21	-0.36	-0,5261 to -0,2037	Yes	****	< 0,0001
4T1 day 14 vs. 4T1 day 21	-6.97	-16,14 to 2,199	No	ns	0.1748	yes	4T1 day 14 vs. 4T1 day 21	-0.16	-0,3156 to -0,004108	Yes	*	0.0427
**aSMA**							**aSMA**					
4T1 Day 7 vs. 4T1 day 14	9.11	-0,3859 to 18,60	No	ns	0.0632	yes	4T1 Day 7 vs. 4T1 day 14	0.36	0,1987 to 0,5210	Yes	****	< 0,0001
4T1 Day 7 vs. 4T1 day 21	11.10	1,603 to 20,59	Yes	*	0.0171		4T1 Day 7 vs. 4T1 day 21	0.42	0,2622 to 0,5846	Yes	****	< 0,0001
4T1 day 14 vs. 4T1 day 21	1.99	-7,182 to 11,16	No	ns	0.8664		4T1 day 14 vs. 4T1 day 21	0.06	-0,09214 to 0,2193	No	ns	0.6022
**CD26**							**CD26**					
4T1 Day 7 vs. 4T1 day 14	-8.33	-17,82 to 1,166	No	ns	0.0989	yes	4T1 Day 7 vs. 4T1 day 14	-0.20	-0,3607 to -0,03828	Yes	*	0.0106
4T1 Day 7 vs. 4T1 day 21	-9.24	-18,73 to 0,2539	No	ns	0.0584	yes	4T1 Day 7 vs. 4T1 day 21	-0.24	-0,4018 to -0,07943	Yes	**	0.0014
4T1 day 14 vs. 4T1 day 21	-0.91	-10,08 to 8,258	No	ns	0.9702		4T1 day 14 vs. 4T1 day 21	-0.04	-0,1969 to 0,1146	No	ns	0.8084
**4T07**							**4T07**					
**PDGFRa**							**PDGFRa**					
4T07 day 7 vs. 4T07 day 14	12.25	2,537 to 21,97	Yes	**	0.009		4T07 day 7 vs. 4T07 day 14	0.25	0,02512 to 0,4834	Yes	*	0.0254
4T07 day 7 vs. 4T07 day 21	5.10	-5,840 to 16,03	No	ns	0.5167		4T07 day 7 vs. 4T07 day 21	0.10	-0,1592 to 0,3565	No	ns	0.6401
4T07 day 14 vs. 4T07 day 21	-7.16	-18,38 to 4,061	No	ns	0.2912		4T07 day 14 vs. 4T07 day 21	-0.16	-0,4201 to 0,1090	No	ns	0.3504
**PDGFRb**							**PDGFRb**					
4T07 day 7 vs. 4T07 day 14	17.08	7,361 to 26,79	Yes	***	0.0001		4T07 day 7 vs. 4T07 day 14	0.31	0,08233 to 0,5406	Yes	**	0.0043
4T07 day 7 vs. 4T07 day 21	15.41	4,473 to 26,34	Yes	**	0.0029		4T07 day 7 vs. 4T07 day 21	0.31	0,04906 to 0,5648	Yes	*	0.0148
4T07 day 14 vs. 4T07 day 21	-1.67	-12,89 to 9,550	No	ns	0.9346		4T07 day 14 vs. 4T07 day 21	0.00	-0,2691 to 0,2600	No	ns	0.9991
**Podoplanin**							**Podoplanin**					
4T07 day 7 vs. 4T07 day 14	14.38	4,664 to 24,10	Yes	**	0.0016		4T07 day 7 vs. 4T07 day 14	0.26	0,02872 to 0,4869	Yes	*	0.0229
4T07 day 7 vs. 4T07 day 21	20.38	9,446 to 31,32	Yes	****	< 0,0001		4T07 day 7 vs. 4T07 day 21	0.38	0,1239 to 0,6396	Yes	**	0.0016
4T07 day 14 vs. 4T07 day 21	6.00	-5,218 to 17,22	No	ns	0.4194		4T07 day 14 vs. 4T07 day 21	0.12	-0,1407 to 0,3884	No	ns	0.5133
**FAP**							**FAP**					
4T07 day 7 vs. 4T07 day 14	-10.50	-20,22 to -0,7858	Yes	*	0.0305	yes	4T07 day 7 vs. 4T07 day 14	-0.22	-0,4513 to 0,006963	No	ns	0.0596
4T07 day 7 vs. 4T07 day 21	-4.48	-15,42 to 6,452	No	ns	0.5994		4T07 day 7 vs. 4T07 day 21	-0.13	-0,3872 to 0,1285	No	ns	0.4655
4T07 day 14 vs. 4T07 day 21	6.02	-5,201 to 17,24	No	ns	0.4173		4T07 day 14 vs. 4T07 day 21	0.09	-0,1717 to 0,3574	No	ns	0.6872
**aSMA**							**aSMA**					
4T07 day 7 vs. 4T07 day 14	10.36	0,6476 to 20,08	Yes	*	0.0334		4T07 day 7 vs. 4T07 day 14	0.34	0,1111 to 0,5694	Yes	**	0.0016
4T07 day 7 vs. 4T07 day 21	11.80	0,8686 to 22,74	Yes	*	0.0308		4T07 day 7 vs. 4T07 day 21	0.42	0,1609 to 0,6766	Yes	***	0.0005
4T07 day 14 vs. 4T07 day 21	1.44	-9,779 to 12,66	No	ns	0.9509		4T07 day 14 vs. 4T07 day 21	0.08	-0,1860 to 0,3431	No	ns	0.7643
**CD26**							**CD26**					
4T07 day 7 vs. 4T07 day 14	4.44	-5,279 to 14,15	No	ns	0.5302		4T07 day 7 vs. 4T07 day 14	0.13	-0,09785 to 0,3604	No	ns	0.3693
4T07 day 7 vs. 4T07 day 21	18.60	7,668 to 29,54	Yes	***	0.0002		4T07 day 7 vs. 4T07 day 21	0.36	0,09721 to 0,6129	Yes	**	0.0037
4T07 day 14 vs. 4T07 day 21	14.17	2,946 to 25,38	Yes	**	0.0089	yes	4T07 day 14 vs. 4T07 day 21	0.22	-0,04075 to 0,4884	No	ns	0.1158

Decrease over time

Increase over time
