# Supplementary material for: Deciphering the temporal heterogeneity of cancer-associated fibroblast subpopulations in breast cancer
Source: J Exp Clin Cancer Res. 2021 May 20;40:175. doi: 10.1186/s13046-021-01944-4 (PMC8138934; doi:10.1186/s13046-021-01944-4)
Supplement: Supplementary file 8 — Additional file 8: Supplementary Table 1. Details of 2-way ANOVA and Tukey’s multiple comparisons post-test on the Lin- population out of live cells reported in main Fig. 2, all repeats combined. Supplementary Table 2. Details of 2-way ANOVA and Tukey’s multiple comparisons post-test on the CAF+ population out of Lin- cells reported in main Fig. 2, all repeats combined. Supplementary Table 3. Details of 2-way ANOVA and Tukey’s multiple comparisons post-test on the CAF+ population out of live cells reported in main Fig. 2, all repeats combined. Supplementary Table 4. Multiple t-tests pertaining to main Fig. 2, all repeats combined. Percentage cells of parent population. Supplementary Table 5. Multiple t-test pertaining to main Fig. 2, all repeats combined. Percentage cells positive for each CAF marker within the CAF+ population. Supplementary Table 6 Tukey’s multiple comparisons from 2-way ANOVA on % of single CAF markers within the CAF+ populations. Data from three independent repeats combined. Significant results reported in main Fig. 2. Supplementary Table 7 Co-expression tables of two CAF markers in 4T1 and 4T07 tumours at different time points, expanding the analysis to contain the time dimension. First CAF marker = parent gate, Subset CAF marker = child gate. CI95 = 95% confidence interval. Supplementary Table 8. Abundance ranking of Boolean CAF subpopulations in 4T1 tumours. Three independent repeats combined, mean % of CAF+ population. Top 5 subpopulations also presented in main Fig. 4. Supplementary Table 9. Abundance ranking of Boolean CAF subpopulations in 4T07 tumours. Three independent repeats combined, mean % of CAF+ population. Top 5 subpopulations also presented in main Fig. 4. Supplementary Table 10. Comparing Boolean CAF subpopulations between tumour types. Percentages from three independent repeats combined. Multiple t-test without assuming equal variance and with FDR correction for multiple comparisons (Q = 1%). Significant results reported in mai [file 13046_2021_1944_MOESM8_ESM.pdf]

table S1

Table Analyzed	Linneg all repeats				
Two-way ANOVA	Ordinary				
Alpha	0.05				
Source of Variation	% of total variation	P value	P value summary	Significant?	
Interaction	2.866	0.1202	ns	No	
day	4.131	0.0482	*	Yes	
tumour type	12.2	< 0,0001	****	Yes	
ANOVA table	SS	DF	MS	F (DFn, DFd)	P value
Interaction	82.99	2	41.49	F (2, 122) = 2,156	P = 0,1202
day	119.6	2	59.81	F (2, 122) = 3,108	P = 0,0482
tumour type	353.4	1	353.4	F (1, 122) = 18,36	P < 0,0001
Residual	2348	122	19.25		

Within each column, compare rows (simple effects within columns)								
Number of families	2							
Number of comparisons per family	3							
Alpha	0.05							
Tukey's multiple comparisons test	Mean Diff,	95% CI of diff,	Significant?	Summary	Adjusted P Value			
4T1								
D7 vs. D14	0.7838	-2,326 to 3,894	No	ns	0.8215			
D7 vs. D21	-2.35	-5,461 to 0,7599	No	ns	0.1762			
D14 vs. D21	-3.134	-6,139 to -0,1294	Yes	*	0.0388			
4T07								
D7 vs. D14	-2.703	-5,813 to 0,4071	No	ns	0.1021			
D7 vs. D21	-2.561	-6,061 to 0,9394	No	ns	0.1961			
D14 vs. D21	0.1421	-3,449 to 3,734	No	ns	0.9952			
Test details	Mean 1	Mean 2	Mean Diff,	SE of diff,	N1	N2	q	DF
4T1								
D7 vs. D14	4.95	4.17	0.78	1.31	21	24	0.8456	122
D7 vs. D21	4.95	7.30	-2.35	1.31	21	24	2.536	122
D14 vs. D21	4.17	7.30	-3.13	1.27	24	24	3.5	122
4T07								
D7 vs. D14	7.11	9.81	-2.70	1.31	24	21	2.916	122
D7 vs. D21	7.11	9.67	-2.56	1.48	24	14	2.455	122
D14 vs. D21	9.81	9.67	0.14	1.51	21	14	0.1328	122

table S2

Table Analyzed	CAF+ out of linneg all repeats				
Two-way ANOVA	Ordinary				
Alpha	0.05				
Source of Variation	% of total variation	P value	P value summary	Significant?	
Interaction	0.32	0.8064	ns	No	
day	10.02	0.0015	**	Yes	
tumour type	1.45	0.1623	ns	No	
ANOVA table	SS	DF	MS	F (DFn, DFd)	P value
Interaction	82.83	2	41.41	F (2, 122) = 0,2155	P = 0,8064
day	2636	2	1318	F (2, 122) = 6,858	P = 0,0015
tumour type	379.9	1	379.9	F (1, 122) = 1,977	P = 0,1623
Residual	23445	122	192.2		

Within each column, compare rows (simple effects within columns)								
Number of families	2							
Number of comparisons per family	3							
Alpha	0.05							
Tukey's multiple comparisons test	Mean Diff,	95% CI of diff,	Significant?	Summary	Adjusted P Value			
4T1								
D7 vs. D14	7.59	-2,239 to 17,42	No	ns	0.1635			
D7 vs. D21	8.45	-1,383 to 18,27	No	ns	0.1073			
D14 vs. D21	0.86	-8,639 to 10,35	No	ns	0.9751			
4T07								
D7 vs. D14	9.69	-0,1382 to 19,52	No	ns	0.0542			
D7 vs. D21	12.52	1,462 to 23,58	Yes	*	0.0223			
D14 vs. D21	2.83	-8,515 to 14,18	No	ns	0.8245			
Test details	Mean 1	Mean 2	Mean Diff,	SE of diff,	N1	N2	q	DF
4T1								
D7 vs. D14	73.14	65.55	7.59	4.14	21	24	2.591	122
D7 vs. D21	73.14	64.70	8.45	4.14	21	24	2.883	122
D14 vs. D21	65.55	64.70	0.86	4.00	24	24	0.3024	122
4T07								
D7 vs. D14	71.69	62.00	9.69	4.14	24	21	3.308	122
D7 vs. D21	71.69	59.17	12.52	4.66	24	14	3.799	122
D14 vs. D21	62.00	59.17	2.83	4.78	21	14	0.8377	122

table S3

Table Analyzed	CAF+ out of live cells all repeats				
Two-way ANOVA	Ordinary				
Alpha	0.05				
**Source of Variation**	**% of total variation**	**P value**	**P value summary**	**Significant?**	
Interaction	2.21	0.2195	ns	No	
day	0.24	0.8467	ns	No	
tumour type	9.14	0.0005	***	Yes	
**ANOVA table**	**SS**	**DF**	**MS**	**F (DFn, DFd)**	**P value**
Interaction	24.46	2	12.23	F (2, 122) = 1,535	P = 0,2195
day	2.655	2	1.327	F (2, 122) = 0,1667	P = 0,8467
tumour type	101.1	1	101.1	F (1, 122) = 12,69	P = 0,0005
Residual	971.7	122	7.965		

Within each column, compare rows (simple effects within columns)								
Number of families	2							
Number of comparisons per family	3							
Alpha	0.05							
**Tukey's multiple comparisons test**	**Mean Diff,**	**95% CI of diff,**	**Significant?**	**Summary**	**Adjusted P Value**			
**4T1**								
D7 vs. D14	0.94	-1,058 to 2,944	No	ns	0.5046			
D7 vs. D21	-0.37	-2,372 to 1,630	No	ns	0.8989			
D14 vs. D21	-1.31	-3,247 to 0,6188	No	ns	0.244			
**4T07**								
D7 vs. D14	-0.82	-2,825 to 1,176	No	ns	0.5923			
D7 vs. D21	-0.21	-2,459 to 2,045	No	ns	0.9741			
D14 vs. D21	0.62	-1,693 to 2,928	No	ns	0.8017			
**Test details**	**Mean 1**	**Mean 2**	**Mean Diff,**	**SE of diff,**	**N1**	**N2**	**q**	**DF**
**4T1**								
D7 vs. D14	3.39	2.45	0.94	0.8433	21	24	1.582	122
D7 vs. D21	3.39	3.76	-0.37	0.8433	21	24	0.6221	122
D14 vs. D21	2.45	3.76	-1.31	0.8147	24	24	2.281	122
**4T07**								
D7 vs. D14	4.67	5.49	-0.82	0.8433	24	21	1.383	122
D7 vs. D21	4.67	4.88	-0.21	0.9491	24	14	0.3086	122
D14 vs. D21	5.49	4.88	0.62	0.9737	21	14	0.8966	122

table S4

Multiple t-tests 4T1 vs 4T07 Lin- out of Live cells								
Day	Discovery?	P value	Mean1 4T1	Mean2 4T07	Difference	SE of difference	t ratio	df
D7		0.039520	4.95	7.11	-2.15	1.01	2.12	43
D14	*	0.000243	4.17	9.81	-5.64	1.41	4.00	43
D21		0.168074	7.30	9.67	-2.36	1.68	1.41	36

Multiple t-tests 4T1 vs 4T07 CAF+ out of Lin- cells								
Day	Discovery?	P value	Mean1 4T1	Mean2 4T07	Difference	SE of difference	t ratio	df
D7		0.761085	73.14	71.69	1.45	4.74	0.31	43
D14		0.273681	65.55	62.00	3.55	3.20	1.11	43
D21		0.268840	64.70	59.17	5.53	4.92	1.12	36

Multiple t-tests 4T1 vs 4T07 CAF+ out of Live cells								
Day	Discovery?	P value	Mean1 4T1	Mean2 4T07	Difference	SE of difference	t ratio	df
D7		0.116181	3.39	4.67	-1.28	0.80	1.60	43
D14	*	0.001006	2.45	5.49	-3.04	0.86	3.53	43
D21		0.266583	3.76	4.88	-1.11	0.99	1.13	36

table S5

Multiple t-tests 4T1 vs 4T07 D7								
CAF marker	Discovery?	P value	Mean1 4T1	Mean2 4T07	Difference	SE of difference	t ratio	df
PDGFRa		0.0363678	48.99	38.28	10.71	4.96	2.16	43
PDGFRb		0.278426	53.38	48.40	4.98	4.54	1.10	43
Podoplanin		0.0041234	55.27	40.49	14.78	4.88	3.03	43
FAP		0.0482698	46.58	51.95	-5.37	2.64	2.03	43
aSMA	*	0.00114589	29.03	19.40	9.63	2.76	3.48	43
CD26		0.0419432	55.93	66.52	-10.58	5.05	2.10	43

Multiple t-tests 4T1 vs 4T07 D14								
CAF marker	Discovery?	P value	Mean1 4T1	Mean2 4T07	Difference	SE of difference	t ratio	df
PDGFRa		0.147984	26.03	19.27	6.76	4.59	1.47	43
PDGFRb		0.0167179	31.32	43.83	-12.51	5.02	2.49	43
Podoplanin		0.0160842	26.11	38.00	-11.89	4.75	2.51	43
FAP		0.0290361	62.45	55.01	7.45	3.30	2.26	43
aSMA	*	0.00013461	9.03	19.92	-10.89	2.60	4.19	43
CD26		0.571223	62.08	64.26	-2.18	3.82	0.57	43

Multiple t-tests 4T1 vs 4T07 D21								
CAF marker	Discovery?	P value	Mean1 4T1	Mean2 4T07	Difference	SE of difference	t ratio	df
PDGFRa	*	0.00138182	18.15	33.19	-15.04	4.34	3.47	36
PDGFRb		0.132781	42.88	32.99	9.89	6.43	1.54	36
Podoplanin		0.0135633	31.49	20.11	11.38	4.38	2.60	36
FAP		0.0904156	61.98	56.44	5.54	3.19	1.74	36
aSMA	*	0.00213947	17.93	7.59	10.34	3.12	3.31	36
CD26	*	8.4759E-06	65.17	47.91	17.26	3.33	5.19	36

table S6

Number of families	6							
Number of comparisons per family	3				Combined			
Alpha	0.05				Repeat 1, 2, 3			
Tukey's multiple comparisons test	Mean Diff,	95% CI of diff,	Significant?	Summary	Adjusted P Value			
PDGFRa								
4T1Day 7 vs. 4T1day 14	29.72	20,23 to 39,22	Yes	****	< 0,0001			
4T1Day 7 vs. 4T1day 21	30.85	21,35 to 40,34	Yes	****	< 0,0001			
4T1day 14 vs. 4T1day 21	1.124	-8,047 to 10,29	No	ns	0.9552			
PDGFRb								
4T1Day 7 vs. 4T1day 14	9.553	0,05991 to 19,05	Yes	*	0.0482			
4T1Day 7 vs. 4T1day 21	10.5	1,011 to 20,00	Yes	*	0.0259			
4T1day 14 vs. 4T1day 21	0.9512	-8,219 to 10,12	No	ns	0.9677			
Podoplanin								
4T1Day 7 vs. 4T1day 14	17.27	7,772 to 26,76	Yes	****	< 0,0001			
4T1Day 7 vs. 4T1day 21	23.78	14,29 to 33,27	Yes	****	< 0,0001			
4T1day 14 vs. 4T1day 21	6.515	-2,656 to 15,69	No	ns	0.2175			
FAP								
4T1Day 7 vs. 4T1day 14	-8.426	-17,92 to 1,067	No	ns	0.0936			
4T1Day 7 vs. 4T1day 21	-15.4	-24,89 to -5,905	Yes	***	0.0005			
4T1day 14 vs. 4T1day 21	-6.972	-16,14 to 2,199	No	ns	0.1748			
aSMA								
4T1Day 7 vs. 4T1day 14	9.107	-0,3859 to 18,60	No	ns	0.0632			
4T1Day 7 vs. 4T1day 21	11.1	1,603 to 20,59	Yes	*	0.0171			
4T1day 14 vs. 4T1day 21	1.989	-7,182 to 11,16	No	ns	0.8664			
CD26								
4T1Day 7 vs. 4T1day 14	-8.326	-17,82 to 1,166	No	ns	0.0989			
4T1Day 7 vs. 4T1day 21	-9.239	-18,73 to 0,2539	No	ns	0.0584			
4T1day 14 vs. 4T1day 21	-0.9125	-10,08 to 8,258	No	ns	0.9702			
Test details	Mean 1	Mean 2	Mean Diff,	SE of diff,	N1	N2	q	DF
PDGFRa								
4T1Day 7 vs. 4T1day 14	48.99	19.27	29.72	4.035	21	24	10.42	396
4T1Day 7 vs. 4T1day 21	48.99	18.15	30.85	4.035	21	24	10.81	396
4T1day 14 vs. 4T1day 21	19.27	18.15	1.124	3.898	24	24	0.4077	396
PDGFRb								
4T1Day 7 vs. 4T1day 14	53.38	43.83	9.553	4.035	21	24	3.348	396
4T1Day 7 vs. 4T1day 21	53.38	42.88	10.5	4.035	21	24	3.681	396
4T1day 14 vs. 4T1day 21	43.83	42.88	0.9512	3.898	24	24	0.3451	396
Podoplanin								
4T1Day 7 vs. 4T1day 14	55.27	38	17.27	4.035	21	24	6.051	396
4T1Day 7 vs. 4T1day 21	55.27	31.49	23.78	4.035	21	24	8.335	396
4T1day 14 vs. 4T1day 21	38	31.49	6.515	3.898	24	24	2.364	396
FAP								
4T1Day 7 vs. 4T1day 14	46.58	55.01	-8.426	4.035	21	24	2.953	396
4T1Day 7 vs. 4T1day 21	46.58	61.98	-15.4	4.035	21	24	5.397	396
4T1day 14 vs. 4T1day 21	55.01	61.98	-6.972	3.898	24	24	2.529	396
aSMA								
4T1Day 7 vs. 4T1day 14	29.03	19.92	9.107	4.035	21	24	3.192	396
4T1Day 7 vs. 4T1day 21	29.03	17.93	11.1	4.035	21	24	3.889	396
4T1day 14 vs. 4T1day 21	19.92	17.93	1.989	3.898	24	24	0.7215	396
CD26								
4T1Day 7 vs. 4T1day 14	55.93	64.26	-8.326	4.035	21	24	2.918	396
4T1Day 7 vs. 4T1day 21	55.93	65.17	-9.239	4.035	21	24	3.238	396
4T1day 14 vs. 4T1day 21	64.26	65.17	-0.9125	3.898	24	24	0.331	396

Number of families	6							
Number of comparisons per family	3				Combined			
Alpha	0.05				Repeat 1, 2, 3			
Tukey's multiple comparisons test	Mean Diff,	95% CI of diff,	Significant?	Summary	Adjusted P Value			
PDGFRa								
4T07 day 7 vs. 4T07 day 14	12.25	2,537 to 21,97	Yes	**	0.009			
4T07 day 7 vs. 4T07 day 21	5.095	-5,840 to 16,03	No	ns	0.5167			
4T07 day 14 vs. 4T07 day 21	-7.158	-18,38 to 4,061	No	ns	0.2912			
PDGFRb								
4T07 day 7 vs. 4T07 day 14	17.08	7,361 to 26,79	Yes	***	0.0001			
4T07 day 7 vs. 4T07 day 21	15.41	4,473 to 26,34	Yes	**	0.0029			
4T07 day 14 vs. 4T07 day 21	-1.669	-12,89 to 9,550	No	ns	0.9346			
Podoplanin								
4T07 day 7 vs. 4T07 day 14	14.38	4,664 to 24,10	Yes	**	0.0016			
4T07 day 7 vs. 4T07 day 21	20.38	9,446 to 31,32	Yes	****	< 0,0001			
4T07 day 14 vs. 4T07 day 21	6.001	-5,218 to 17,22	No	ns	0.4194			
FAP								
4T07 day 7 vs. 4T07 day 14	-10.5	-20,22 to -0,7858	Yes	*	0.0305			
4T07 day 7 vs. 4T07 day 21	-4.484	-15,42 to 6,452	No	ns	0.5994			
4T07 day 14 vs. 4T07 day 21	6.018	-5,201 to 17,24	No	ns	0.4173			
aSMA								
4T07 day 7 vs. 4T07 day 14	10.36	0,6476 to 20,08	Yes	*	0.0334			
4T07 day 7 vs. 4T07 day 21	11.8	0,8686 to 22,74	Yes	*	0.0308			
4T07 day 14 vs. 4T07 day 21	1.44	-9,779 to 12,66	No	ns	0.9509			
CD26								
4T07 day 7 vs. 4T07 day 14	4.437	-5,279 to 14,15	No	ns	0.5302			
4T07 day 7 vs. 4T07 day 21	18.6	7,668 to 29,54	Yes	***	0.0002			
4T07 day 14 vs. 4T07 day 21	14.17	2,946 to 25,38	Yes	**	0.0089			
Test details	Mean 1	Mean 2	Mean Diff,	SE of diff,	N1	N2	q	DF
PDGFRa								
4T07 day 7 vs. 4T07 day 14	38.28	26.03	12.25	4.127	24	21	4.199	336
4T07 day 7 vs. 4T07 day 21	38.28	33.19	5.095	4.645	24	14	1.551	336
4T07 day 14 vs. 4T07 day 21	26.03	33.19	-7.158	4.766	21	14	2.124	336
PDGFRb								
4T07 day 7 vs. 4T07 day 14	48.4	31.32	17.08	4.127	24	21	5.852	336
4T07 day 7 vs. 4T07 day 21	48.4	32.99	15.41	4.645	24	14	4.691	336
4T07 day 14 vs. 4T07 day 21	31.32	32.99	-1.669	4.766	21	14	0.4953	336
Podoplanin								
4T07 day 7 vs. 4T07 day 14	40.49	26.11	14.38	4.127	24	21	4.928	336
4T07 day 7 vs. 4T07 day 21	40.49	20.11	20.38	4.645	24	14	6.205	336
4T07 day 14 vs. 4T07 day 21	26.11	20.11	6.001	4.766	21	14	1.781	336
FAP								
4T07 day 7 vs. 4T07 day 14	51.95	62.45	-10.5	4.127	24	21	3.599	336
4T07 day 7 vs. 4T07 day 21	51.95	56.44	-4.484	4.645	24	14	1.365	336
4T07 day 14 vs. 4T07 day 21	62.45	56.44	6.018	4.766	21	14	1.786	336
aSMA								
4T07 day 7 vs. 4T07 day 14	19.4	9.034	10.36	4.127	24	21	3.551	336
4T07 day 7 vs. 4T07 day 21	19.4	7.594	11.8	4.645	24	14	3.594	336
4T07 day 14 vs. 4T07 day 21	9.034	7.594	1.44	4.766	21	14	0.4273	336
CD26								
4T07 day 7 vs. 4T07 day 14	66.52	62.08	4.437	4.127	24	21	1.521	336
4T07 day 7 vs. 4T07 day 21	66.52	47.91	18.6	4.645	24	14	5.664	336
4T07 day 14 vs. 4T07 day 21	62.08	47.91	14.17	4.766	21	14	4.204	336

table S7

4T1

Day 7	① First CAF marker																	
② Subset CAF marker	PDGFRa			PDGFRb			Podoplanin			FAPa			aSMA			CD26		
	Mean	$CI95_{low}$	$CI95_{high}$	Mean	$CI95_{low}$	$CI95_{high}$	Mean	$CI95_{low}$	$CI95_{high}$	Mean	$CI95_{low}$	$CI95_{high}$	Mean	$CI95_{low}$	$CI95_{high}$	Mean	$CI95_{low}$	$CI95_{high}$
PDGFRa	100.00	100.00	100.00	50.67	43.01	58.33	65.14	59.10	71.18	38.09	29.13	47.05	49.81	42.43	57.19	45.86	36.29	55.43
PDGFRb	58.00	50.04	65.96	100.00	100.00	100.00	68.52	61.14	75.90	62.02	57.46	66.57	71.93	64.62	79.24	60.66	53.67	67.65
Podoplanin	77.70	68.49	86.91	68.42	63.67	73.16	100.00	100.00	100.00	41.52	32.15	50.88	88.92	84.20	93.64	64.04	55.37	72.70
FAPa	34.55	29.23	39.87	55.69	49.30	62.07	34.26	28.46	40.05	100.00	100.00	100.00	26.32	19.64	32.99	49.32	46.41	52.23
aSMA	31.62	25.84	37.40	37.84	33.57	42.11	47.37	41.18	53.56	16.07	11.27	20.86	100.00	100.00	100.00	33.11	26.95	39.27
CD26	52.88	42.70	63.05	62.00	55.79	68.22	63.40	55.97	70.82	59.04	51.98	66.10	60.70	53.69	67.70	100.00	100.00	100.00

Day 14	① First CAF marker																	
② Subset CAF marker	PDGFRa			PDGFRb			Podoplanin			FAPa			aSMA			CD26		
	Mean	$CI95_{low}$	$CI95_{high}$	Mean	$CI95_{low}$	$CI95_{high}$	Mean	$CI95_{low}$	$CI95_{high}$	Mean	$CI95_{low}$	$CI95_{high}$	Mean	$CI95_{low}$	$CI95_{high}$	Mean	$CI95_{low}$	$CI95_{high}$
PDGFRa	100.00	100.00	100.00	26.67	19.45	33.89	40.34	33.42	47.26	15.06	10.63	19.50	25.95	20.65	31.24	22.00	14.83	29.18
PDGFRb	62.74	56.76	68.71	100.00	100.00	100.00	66.43	60.65	72.20	52.21	45.06	59.35	70.22	65.81	74.62	46.62	38.56	54.67
Podoplanin	84.31	79.56	89.06	55.06	47.71	62.41	100.00	100.00	100.00	27.29	21.02	33.56	88.67	86.59	90.74	43.91	34.39	53.44
FAPa	46.95	39.76	54.14	67.37	62.77	71.98	43.09	35.59	50.60	100.00	100.00	100.00	31.62	24.98	38.26	49.72	45.16	54.28
aSMA	27.58	23.21	31.95	29.62	25.50	33.74	46.06	39.87	52.25	10.12	7.19	13.04	100.00	100.00	100.00	19.87	15.27	24.47
CD26	71.69	66.72	76.65	67.91	63.38	72.44	71.83	66.91	76.76	57.34	54.43	60.25	62.95	58.42	67.49	100.00	100.00	100.00

Day 21	① First CAF marker																	
② Subset CAF marker	PDGFRa			PDGFRb			Podoplanin			FAPa			aSMA			CD26		
	Mean	$CI95_{low}$	$CI95_{high}$	Mean	$CI95_{low}$	$CI95_{high}$	Mean	$CI95_{low}$	$CI95_{high}$	Mean	$CI95_{low}$	$CI95_{high}$	Mean	$CI95_{low}$	$CI95_{high}$	Mean	$CI95_{low}$	$CI95_{high}$
PDGFRa	100.00	100.00	100.00	26.74	20.16	33.31	42.85	38.55	47.15	13.88	10.64	17.13	26.07	22.21	29.94	20.66	15.69	25.63
PDGFRb	56.32	48.39	64.25	100.00	100.00	100.00	66.96	60.11	73.80	47.74	39.82	55.67	71.59	64.28	78.91	46.83	38.40	55.26
Podoplanin	74.90	65.76	84.03	51.43	42.87	59.99	100.00	100.00	100.00	23.23	17.89	28.58	87.63	84.68	90.58	36.34	29.21	43.47
FAPa	47.46	41.19	53.73	70.59	65.03	76.14	47.77	39.65	55.89	100.00	100.00	100.00	36.46	29.18	43.74	57.52	54.11	60.92
aSMA	25.58	19.26	31.90	29.82	22.81	36.84	45.66	40.75	50.58	10.53	6.54	14.52	100.00	100.00	100.00	18.16	13.45	22.87
CD26	70.56	63.85	77.28	71.27	67.12	75.43	73.88	71.09	76.66	60.35	57.60	63.11	66.14	62.42	69.86	100.00	100.00	100.00

4T07

Day 7	① First CAF marker																	
② Subset CAF marker	PDGFRa			PDGFRb			Podoplanin			FAPa			aSMA			CD26		
	Mean	$CI95_{low}$	$CI95_{high}$	Mean	$CI95_{low}$	$CI95_{high}$	Mean	$CI95_{low}$	$CI95_{high}$	Mean	$CI95_{low}$	$CI95_{high}$	Mean	$CI95_{low}$	$CI95_{high}$	Mean	$CI95_{low}$	$CI95_{high}$
PDGFRa	100.00	100.00	100.00	46.06	39.96	52.15	65.84	61.33	70.35	29.68	24.32	35.04	51.11	45.54	56.68	35.77	29.76	41.77
PDGFRb	60.93	53.01	68.86	100.00	100.00	100.00	73.94	69.47	78.40	57.16	52.16	62.15	80.34	76.63	84.05	54.01	47.07	60.95
Podoplanin	75.81	65.84	85.79	60.42	55.34	65.51	100.00	100.00	100.00	34.01	29.30	38.71	88.01	83.96	92.06	48.71	41.78	55.65
FAPa	41.09	36.47	45.72	63.80	58.82	68.78	46.81	40.94	52.68	100.00	100.00	100.00	38.05	31.57	44.54	51.10	48.16	54.05
aSMA	29.10	23.46	34.75	31.99	27.61	36.38	42.25	37.88	46.62	13.47	10.43	16.51	100.00	100.00	100.00	22.20	18.48	25.93
CD26	66.62	55.90	77.33	72.07	67.52	76.61	78.21	73.32	83.11	63.88	58.34	69.42	74.91	69.13	80.70	100.00	100.00	100.00

Day 14	① First CAF marker																	
② Subset CAF marker	PDGFRa			PDGFRb			Podoplanin			FAPa			aSMA			CD26		
	Mean	$CI95_{low}$	$CI95_{high}$	Mean	$CI95_{low}$	$CI95_{high}$	Mean	$CI95_{low}$	$CI95_{high}$	Mean	$CI95_{low}$	$CI95_{high}$	Mean	$CI95_{low}$	$CI95_{high}$	Mean	$CI95_{low}$	$CI95_{high}$
PDGFRa	100.00	100.00	100.00	35.67	28.90	42.44	58.62	52.58	64.65	19.44	13.47	25.41	50.10	44.13	56.07	23.38	17.04	29.71
PDGFRb	43.33	36.41	50.25	100.00	100.00	100.00	54.13	47.46	60.79	38.53	31.06	46.00	66.19	61.04	71.34	36.84	28.09	45.58
Podoplanin	66.72	56.53	76.90	46.78	39.87	53.69	100.00	100.00	100.00	22.76	17.44	28.08	88.07	83.57	92.58	31.27	24.02	38.52
FAPa	45.55	39.42	51.68	78.53	74.81	82.25	54.76	49.91	59.61	100.00	100.00	100.00	50.95	44.35	57.54	57.50	51.22	63.78
aSMA	19.89	15.78	24.00	19.99	16.28	23.69	30.66	25.92	35.40	7.11	5.24	8.98	100.00	100.00	100.00	9.73	7.29	12.18
CD26	62.39	50.86	73.91	71.55	66.32	76.78	73.89	66.94	80.83	55.90	50.75	61.05	66.29	59.84	72.74	100.00	100.00	100.00

Day 21	① First CAF marker																	
② Subset CAF marker	PDGFRa			PDGFRb			Podoplanin			FAPa			aSMA			CD26		
	Mean	$CI95_{low}$	$CI95_{high}$	Mean	$CI95_{low}$	$CI95_{high}$	Mean	$CI95_{low}$	$CI95_{high}$	Mean	$CI95_{low}$	$CI95_{high}$	Mean	$CI95_{low}$	$CI95_{high}$	Mean	$CI95_{low}$	$CI95_{high}$
PDGFRa	100.00	100.00	100.00	34.42	24.70	44.14	59.55	51.54	67.57	20.27	13.43	27.10	47.31	41.62	53.00	25.21	16.60	33.82
PDGFRb	38.98	25.05	52.91	100.00	100.00	100.00	58.09	47.43	68.74	40.88	30.98	50.77	65.45	52.47	78.43	37.64	26.88	48.40
Podoplanin	45.10	29.38	60.82	38.22	28.35	48.09	100.00	100.00	100.00	15.72	11.82	19.63	82.57	71.31	93.84	24.73	17.50	31.96
FAPa	34.45	28.43	40.47	75.58	68.22	82.94	48.50	40.28	56.71	100.00	100.00	100.00	35.98	27.41	44.55	56.77	50.52	63.02
aSMA	14.22	7.99	20.45	15.49	10.16	20.82	29.70	21.98	37.42	4.16	2.67	5.65	100.00	100.00	100.00	6.84	4.53	9.15
CD26	42.43	29.01	55.85	56.74	49.29	64.18	59.70	51.50	67.90	47.12	43.03	51.21	46.53	37.30	55.76	100.00	100.00	100.00

table S8

	4T1 D7				4T1 D14				4T1 D21			
Top	CAF subset	Mean %	SEM	n	CAF subset	Mean %	SEM	n	CAF subset	Mean %	SEM	n
1	S56	8.07	1.17	21	S32	16.45	3.13	24	S32	15.09	1.96	24
2	S63	7.07	2.14	21	S56	13.98	1.34	24	S56	14.55	0.76	24
3	S22	6.87	0.85	21	S22	10.38	1.31	24	S22	13.21	2.03	24
4	S32	5.84	1.33	21	S24	8.11	0.96	24	S24	11.35	1.23	24
5	S17	5.82	0.88	21	S54	6.41	0.95	24	S54	6.19	1.25	24
6	S27	4.83	1.20	21	S10	3.84	0.60	24	S10	2.97	0.52	24
7	S54	4.45	0.48	21	S27	3.78	1.06	24	S17	2.72	0.32	24
8	S9	4.20	0.63	21	S17	3.39	0.51	24	S18	2.09	0.28	24
9	S10	3.78	0.59	21	S42	2.72	0.57	24	S25	2.02	0.38	24
10	S41	3.56	0.71	21	S18	2.49	0.18	24	S2	1.97	0.21	24
11	S1	3.55	0.75	21	S12	2.07	0.32	24	S27	1.92	0.43	24
12	S24	3.24	0.61	21	S2	1.94	0.24	24	S42	1.79	0.33	24
13	S57	3.23	0.44	21	S25	1.92	0.34	24	S1	1.66	0.35	24
14	S25	2.59	0.31	21	S1	1.66	0.29	24	S9	1.50	0.24	24
15	S42	2.44	0.44	21	S62	1.66	0.25	24	S62	1.47	0.14	24
16	S59	2.15	0.31	21	S44	1.47	0.36	24	S63	1.45	0.34	24
17	S62	2.06	0.30	21	S28	1.46	0.31	24	S12	1.35	0.23	24
18	S19	2.02	0.53	21	S63	1.40	0.65	24	S20	1.17	0.17	24
19	S12	1.95	0.37	21	S9	1.36	0.17	24	S44	1.13	0.25	24
20	S11	1.92	0.45	21	S19	1.14	0.28	24	S19	1.10	0.21	24
21	S18	1.74	0.17	21	S20	1.05	0.17	24	S48	1.06	0.27	24
22	S2	1.40	0.18	21	S48	0.90	0.14	24	S55	0.82	0.23	24
23	S49	1.28	0.12	21	S26	0.85	0.12	24	S60	0.69	0.10	24
24	S55	1.27	0.44	21	S41	0.74	0.15	24	S28	0.64	0.08	24
25	S48	1.16	0.22	21	S50	0.70	0.06	24	S26	0.64	0.11	24
26	S21	1.04	0.18	21	S58	0.67	0.11	24	S11	0.59	0.12	24
27	S28	1.00	0.21	21	S57	0.65	0.11	24	S23	0.57	0.08	24
28	S58	0.95	0.17	21	S60	0.62	0.05	24	S52	0.55	0.08	24
29	S44	0.81	0.09	21	S11	0.58	0.16	24	S41	0.53	0.08	24
30	S26	0.71	0.09	21	S52	0.53	0.08	24	S50	0.53	0.05	24
31	S20	0.70	0.12	21	S34	0.48	0.05	24	S57	0.52	0.08	24
32	S43	0.67	0.08	21	S46	0.45	0.07	24	S58	0.50	0.09	24
33	S50	0.61	0.09	21	S59	0.44	0.07	24	S4	0.46	0.14	24
34	S3	0.59	0.20	21	S55	0.44	0.12	24	S59	0.46	0.10	24
35	S60	0.58	0.06	21	S30	0.38	0.05	24	S34	0.45	0.06	24
36	S53	0.57	0.10	21	S4	0.32	0.08	24	S30	0.43	0.04	24
37	S33	0.51	0.06	21	S49	0.31	0.03	24	S21	0.38	0.06	24
38	S46	0.51	0.09	21	S21	0.26	0.03	24	S31	0.37	0.18	24
39	S31	0.50	0.14	21	S40	0.23	0.05	24	S40	0.31	0.09	24
40	S23	0.44	0.08	21	S36	0.19	0.05	24	S49	0.31	0.03	24
41	S4	0.40	0.13	21	S43	0.18	0.04	24	S3	0.29	0.09	24
42	S51	0.39	0.05	21	S23	0.17	0.03	24	S36	0.28	0.09	24
43	S52	0.37	0.09	21	S3	0.16	0.05	24	S43	0.25	0.07	24
44	S34	0.35	0.05	21	S31	0.16	0.06	24	S46	0.24	0.03	24
45	S61	0.34	0.07	21	S33	0.16	0.03	24	S33	0.22	0.06	24
46	S30	0.19	0.03	21	S51	0.10	0.02	24	S16	0.21	0.06	24
47	S6	0.18	0.04	21	S8	0.09	0.03	24	S8	0.21	0.05	24
48	S38	0.16	0.04	21	S53	0.09	0.01	24	S6	0.15	0.02	24
49	S40	0.16	0.04	21	S16	0.09	0.02	24	S51	0.13	0.03	24
50	S36	0.11	0.02	21	S38	0.08	0.01	24	S38	0.11	0.02	24
51	S35	0.10	0.02	21	S6	0.08	0.01	24	S53	0.10	0.02	24
52	S8	0.10	0.03	21	S61	0.08	0.03	24	S61	0.07	0.02	24
53	S29	0.08	0.02	21	S14	0.05	0.01	24	S35	0.05	0.02	24
54	S14	0.08	0.02	21	S35	0.03	0.01	24	S29	0.04	0.01	24
55	S16	0.07	0.01	21	S29	0.02	0.00	24	S14	0.03	0.01	24
56	S45	0.07	0.02	21	S45	0.01	0.00	24	S47	0.03	0.01	24
57	S5	0.07	0.02	21	S5	0.01	0.00	24	S5	0.02	0.01	24
58	S47	0.06	0.01	21	S47	0.01	0.00	24	S37	0.01	0.00	24
59	S7	0.03	0.01	21	S37	0.00	0.00	24	S39	0.01	0.00	24
60	S15	0.03	0.01	21	S39	0.00	0.00	24	S7	0.01	0.00	24
61	S37	0.02	0.01	21	S7	0.00	0.00	24	S45	0.01	0.00	24
62	S39	0.02	0.01	21	S15	0.00	0.00	24	S15	0.01	0.00	24
63	S13	0.01	0.00	21	S13	0.00	0.00	24	S13	0.00	0.00	24

table S9

	4T07 D7					4T07 D14					4T07 D21			
Top	CAF subset	Mean %	SEM	n		CAF subset	Mean %	SEM	n		CAF subset	Mean %	SEM	n
1	S32	14.44	1.94	24		S56	17.73	1.08	21		S56	16.88	1.21	14
2	S56	9.48	0.82	24		S32	17.32	2.50	21		S32	13.72	2.49	14
3	S22	9.39	1.05	24		S24	12.32	1.61	21		S63	13.69	4.41	14
4	S24	7.23	0.94	24		S22	9.88	1.43	21		S24	8.95	1.49	14
5	S63	6.49	2.62	24		S63	6.03	3.02	21		S22	8.91	1.89	14
6	S17	5.33	0.48	24		S54	4.70	1.02	21		S54	7.66	1.82	14
7	S54	4.52	0.52	24		S27	3.56	0.84	21		S27	2.45	0.72	14
8	S9	4.48	0.60	24		S17	3.29	0.56	21		S55	2.31	0.70	14
9	S25	3.55	0.52	24		S19	1.94	0.42	21		S17	2.10	0.42	14
10	S1	3.15	0.59	24		S18	1.88	0.24	21		S62	1.72	0.40	14
11	S10	2.97	0.57	24		S1	1.59	0.30	21		S21	1.17	0.24	14
12	S27	2.96	0.52	24		S55	1.52	0.48	21		S25	1.13	0.34	14
13	S18	2.07	0.21	24		S20	1.33	0.19	21		S19	1.12	0.29	14
14	S2	1.78	0.16	24		S25	1.16	0.18	21		S31	1.06	0.41	14
15	S57	1.70	0.31	24		S2	1.09	0.15	21		S23	1.02	0.20	14
16	S19	1.52	0.22	24		S28	0.97	0.22	21		S42	0.99	0.37	14
17	S62	1.36	0.12	24		S62	0.89	0.16	21		S18	0.91	0.11	14
18	S55	1.18	0.46	24		S9	0.88	0.17	21		S41	0.84	0.20	14
19	S41	1.04	0.20	24		S52	0.71	0.10	21		S61	0.80	0.18	14
20	S59	1.02	0.17	24		S21	0.69	0.19	21		S1	0.76	0.17	14
21	S11	0.94	0.19	24		S11	0.61	0.14	21		S59	0.71	0.17	14
22	S28	0.88	0.13	24		S60	0.57	0.10	21		S9	0.71	0.24	14
23	S26	0.87	0.19	24		S10	0.56	0.13	21		S50	0.68	0.13	14
24	S49	0.77	0.09	24		S41	0.55	0.11	21		S20	0.66	0.10	14
25	S21	0.76	0.09	24		S42	0.55	0.16	21		S57	0.63	0.12	14
26	S42	0.74	0.12	24		S50	0.54	0.12	21		S44	0.62	0.20	14
27	S20	0.72	0.09	24		S23	0.53	0.08	21		S60	0.62	0.14	14
28	S12	0.69	0.11	24		S31	0.48	0.14	21		S48	0.59	0.27	14
29	S48	0.66	0.12	24		S3	0.45	0.12	21		S49	0.58	0.10	14
30	S23	0.62	0.08	24		S57	0.44	0.08	21		S58	0.58	0.20	14
31	S53	0.52	0.17	24		S59	0.43	0.07	21		S28	0.57	0.17	14
32	S61	0.51	0.11	24		S12	0.36	0.06	21		S53	0.56	0.11	14
33	S58	0.44	0.10	24		S61	0.36	0.09	21		S10	0.53	0.21	14
34	S30	0.43	0.07	24		S44	0.35	0.05	21		S2	0.39	0.08	14
35	S31	0.43	0.09	24		S49	0.35	0.06	21		S11	0.33	0.10	14
36	S50	0.41	0.06	24		S34	0.31	0.05	21		S52	0.32	0.06	14
37	S6	0.40	0.15	24		S48	0.29	0.07	21		S51	0.31	0.11	14
38	S60	0.35	0.07	24		S51	0.28	0.08	21		S26	0.29	0.10	14
39	S51	0.31	0.07	24		S30	0.27	0.06	21		S12	0.25	0.07	14
40	S3	0.27	0.06	24		S26	0.26	0.05	21		S34	0.24	0.06	14
41	S46	0.27	0.03	24		S4	0.25	0.07	21		S30	0.23	0.06	14
42	S44	0.26	0.05	24		S53	0.24	0.04	21		S33	0.21	0.05	14
43	S33	0.25	0.03	24		S58	0.23	0.05	21		S43	0.20	0.06	14
44	S34	0.24	0.03	24		S33	0.23	0.04	21		S3	0.19	0.10	14
45	S4	0.22	0.05	24		S43	0.20	0.04	21		S29	0.09	0.05	14
46	S52	0.22	0.03	24		S6	0.16	0.07	21		S46	0.09	0.02	14
47	S43	0.22	0.04	24		S36	0.12	0.02	21		S4	0.09	0.03	14
48	S38	0.18	0.08	24		S40	0.08	0.01	21		S36	0.08	0.03	14
49	S40	0.17	0.04	24		S8	0.08	0.02	21		S6	0.07	0.02	14
50	S29	0.14	0.03	24		S29	0.07	0.02	21		S7	0.06	0.06	14
51	S8	0.09	0.02	24		S38	0.07	0.02	21		S38	0.05	0.02	14
52	S16	0.08	0.01	24		S35	0.06	0.02	21		S40	0.05	0.02	14
53	S36	0.08	0.01	24		S46	0.06	0.02	21		S47	0.05	0.03	14
54	S14	0.06	0.01	24		S16	0.05	0.02	21		S5	0.05	0.03	14
55	S35	0.04	0.01	24		S47	0.02	0.01	21		S16	0.04	0.02	14
56	S45	0.03	0.01	24		S39	0.01	0.01	21		S8	0.04	0.01	14
57	S47	0.02	0.00	24		S14	0.01	0.00	21		S35	0.02	0.01	14
58	S5	0.02	0.00	24		S7	0.01	0.00	21		S14	0.02	0.01	14
59	S15	0.01	0.00	24		S5	0.01	0.00	21		S45	0.02	0.00	14
60	S13	0.01	0.00	24		S15	0.01	0.00	21		S15	0.01	0.01	14
61	S37	0.01	0.00	24		S45	0.01	0.00	21		S39	0.01	0.00	14
62	S7	0.01	0.00	24		S13	0.00	0.00	21		S37	0.01	0.00	14
63	S39	0.00	0.00	24		S37	0.00	0.00	21		S13	0.00	0.00	14

table S10

Multiple t-tests 4T1 vs 4T07 D7

CAF subset	Discovery?	P value	Mean1 4T1	Mean2 4T07	Difference	SE of difference	t ratio	df
S1		0.670128	3.55	3.15	0.41	0.94	0.429	43
S2		0.115340	1.40	1.78	-0.38	0.24	1.607	43
S3		0.113867	0.59	0.27	0.31	0.19	1.614	43
S4		0.197771	0.40	0.22	0.17	0.13	1.308	43
S5		0.006119	0.07	0.02	0.05	0.02	2.884	43
S6		0.194937	0.18	0.40	-0.22	0.17	1.317	43
S7		0.018639	0.03	0.01	0.02	0.01	2.445	43
S8		0.775064	0.10	0.09	0.01	0.03	0.288	43
S9		0.750949	4.20	4.48	-0.28	0.87	0.319	43
S10		0.332852	3.78	2.97	0.80	0.82	0.979	43
S11		0.041256	1.92	0.94	0.98	0.47	2.104	43
S12	*	0.001220	1.95	0.69	1.26	0.36	3.463	43
S13		0.708238	0.01	0.01	0.00	0.01	0.377	43
S14		0.384671	0.08	0.06	0.02	0.02	0.878	43
S15		0.149474	0.03	0.01	0.01	0.01	1.468	43
S16		0.574243	0.07	0.08	-0.01	0.02	0.566	43
S17		0.620748	5.82	5.33	0.48	0.97	0.498	43
S18		0.237856	1.74	2.07	-0.33	0.28	1.197	43
S19		0.369887	2.02	1.52	0.50	0.55	0.906	43
S20		0.871153	0.70	0.72	-0.02	0.15	0.163	43
S21		0.159622	1.04	0.76	0.28	0.20	1.431	43
S22		0.072977	6.87	9.39	-2.53	1.37	1.838	43
S23		0.123313	0.44	0.62	-0.17	0.11	1.572	43
S24	*	0.001268	3.24	7.23	-3.99	1.16	3.450	43
S25		0.134095	2.59	3.55	-0.96	0.63	1.527	43
S26		0.463034	0.71	0.87	-0.16	0.22	0.740	43
S27		0.142609	4.83	2.96	1.87	1.25	1.494	43
S28		0.631965	1.00	0.88	0.12	0.24	0.482	43
S29		0.085995	0.08	0.14	-0.06	0.03	1.757	43
S30		0.006813	0.19	0.43	-0.23	0.08	2.843	43
S31		0.665055	0.50	0.43	0.07	0.16	0.436	43
S32	*	0.000918	5.84	14.44	-8.60	2.41	3.561	43
S33	*	0.000139	0.51	0.25	0.26	0.06	4.183	43
S34		0.082846	0.35	0.24	0.10	0.06	1.776	43
S35		0.015673	0.10	0.04	0.06	0.02	2.516	43
S36		0.203425	0.11	0.08	0.03	0.02	1.292	43
S37		0.032380	0.02	0.01	0.01	0.01	2.211	43
S38		0.782050	0.16	0.18	-0.03	0.10	0.278	43
S39		0.039842	0.02	0.00	0.01	0.01	2.120	43
S40		0.788940	0.16	0.17	-0.02	0.06	0.269	43
S41	*	0.000789	3.56	1.04	2.52	0.70	3.612	43
S42	*	0.000322	2.44	0.74	1.69	0.43	3.911	43
S43	*	0.000008	0.67	0.22	0.45	0.09	5.074	43
S44	*	0.000001	0.81	0.26	0.55	0.10	5.590	43
S45		0.044907	0.07	0.03	0.04	0.02	2.066	43
S46		0.010461	0.51	0.27	0.24	0.09	2.677	43
S47		0.002508	0.06	0.02	0.03	0.01	3.211	43
S48		0.043457	1.16	0.66	0.51	0.24	2.081	43
S49	*	0.001231	1.28	0.77	0.51	0.15	3.460	43
S50		0.050778	0.61	0.41	0.20	0.10	2.010	43
S51		0.388689	0.39	0.31	0.08	0.09	0.871	43
S52		0.097134	0.37	0.22	0.15	0.09	1.696	43
S53		0.785562	0.57	0.52	0.06	0.21	0.274	43
S54		0.914057	4.45	4.52	-0.08	0.71	0.109	43
S55		0.893838	1.27	1.18	0.09	0.64	0.134	43
S56		0.322404	8.07	9.48	-1.40	1.40	1.001	43
S57		0.006089	3.23	1.70	1.53	0.53	2.885	43
S58		0.010897	0.95	0.44	0.51	0.19	2.661	43
S59		0.002165	2.15	1.02	1.13	0.35	3.263	43
S60		0.017022	0.58	0.35	0.23	0.09	2.483	43
S61		0.207491	0.34	0.51	-0.17	0.14	1.280	43
S62		0.031101	2.06	1.36	0.70	0.31	2.229	43
S63		0.868287	7.07	6.49	0.57	3.45	0.167	43

Multiple t-tests 4T1 vs 4T07 D14

CAF subset	Discovery?	P value	Mean1 4T1	Mean2 4T07	Difference	SE of difference	t ratio	df
S1		0.862189	1.66	1.59	0.07	0.42	0.175	43
S2		0.005825	1.94	1.09	0.85	0.29	2.902	43
S3		0.018291	0.16	0.45	-0.29	0.12	2.453	43
S4		0.521111	0.32	0.25	0.07	0.11	0.647	43
S5		0.641022	0.01	0.01	0.00	0.00	0.470	43
S6		0.225144	0.08	0.16	-0.08	0.07	1.231	43
S7		0.066966	0.00	0.01	-0.01	0.00	1.879	43
S8		0.672961	0.09	0.08	0.02	0.04	0.425	43
S9		0.053233	1.36	0.88	0.48	0.24	1.988	43
S10	*	0.000009	3.84	0.56	3.28	0.65	5.047	43
S11		0.899971	0.58	0.61	-0.03	0.22	0.126	43
S12	*	0.000014	2.07	0.36	1.70	0.35	4.905	43
S13		0.439272	0.00	0.00	0.00	0.00	0.781	43
S14	*	0.000159	0.05	0.01	0.04	0.01	4.140	43
S15		0.216424	0.00	0.01	0.00	0.00	1.255	43
S16		0.192132	0.09	0.05	0.04	0.03	1.325	43
S17		0.894037	3.39	3.29	0.10	0.76	0.134	43
S18		0.042731	2.49	1.88	0.62	0.30	2.088	43
S19		0.108571	1.14	1.94	-0.80	0.49	1.639	43
S20		0.285773	1.05	1.33	-0.28	0.26	1.081	43
S21		0.022461	0.26	0.69	-0.43	0.18	2.368	43
S22		0.798111	10.38	9.88	0.50	1.94	0.257	43
S23	*	0.000080	0.17	0.53	-0.36	0.08	4.359	43
S24		0.025527	8.11	12.32	-4.21	1.82	2.314	43
S25		0.069355	1.92	1.16	0.76	0.41	1.863	43
S26	*	0.000081	0.85	0.26	0.59	0.13	4.356	43
S27		0.870810	3.78	3.56	0.23	1.38	0.164	43
S28		0.218536	1.46	0.97	0.49	0.39	1.249	43
S29		0.002540	0.02	0.07	-0.05	0.02	3.206	43
S30		0.148259	0.38	0.27	0.11	0.07	1.472	43
S31		0.030607	0.16	0.48	-0.32	0.14	2.236	43
S32		0.832101	16.45	17.32	-0.87	4.08	0.213	43
S33		0.119713	0.16	0.23	-0.08	0.05	1.588	43
S34		0.018420	0.48	0.31	0.17	0.07	2.450	43
S35		0.194486	0.03	0.06	-0.03	0.02	1.318	43
S36		0.235245	0.19	0.12	0.07	0.06	1.204	43
S37		0.202029	0.00	0.00	0.00	0.00	1.296	43
S38		0.477959	0.08	0.07	0.01	0.02	0.716	43
S39		0.195444	0.00	0.01	-0.01	0.01	1.315	43
S40		0.013852	0.23	0.08	0.15	0.06	2.566	43
S41		0.321236	0.74	0.55	0.19	0.19	1.003	43
S42	*	0.001271	2.72	0.55	2.18	0.63	3.449	43
S43		0.743423	0.18	0.20	-0.02	0.05	0.329	43
S44		0.006553	1.47	0.35	1.12	0.39	2.858	43
S45		0.225895	0.01	0.01	0.01	0.00	1.229	43
S46	*	0.000007	0.45	0.06	0.39	0.08	5.109	43
S47		0.046365	0.01	0.02	-0.02	0.01	2.051	43
S48	*	0.000469	0.90	0.29	0.61	0.16	3.787	43
S49		0.618470	0.31	0.35	-0.03	0.06	0.502	43
S50		0.229197	0.70	0.54	0.16	0.13	1.220	43
S51		0.027193	0.10	0.28	-0.18	0.08	2.287	43
S52		0.165022	0.53	0.71	-0.18	0.13	1.412	43
S53	*	0.001104	0.09	0.24	-0.15	0.04	3.498	43
S54		0.226255	6.41	4.70	1.71	1.40	1.228	43
S55		0.025723	0.44	1.52	-1.08	0.47	2.311	43
S56		0.038064	13.98	17.73	-3.74	1.75	2.140	43
S57		0.149292	0.65	0.44	0.20	0.14	1.468	43
S58	*	0.001065	0.67	0.23	0.43	0.12	3.510	43
S59		0.926590	0.44	0.43	0.01	0.10	0.093	43
S60		0.615706	0.62	0.57	0.05	0.10	0.506	43
S61		0.002566	0.08	0.36	-0.28	0.09	3.202	43
S62		0.014361	1.66	0.89	0.77	0.30	2.552	43
S63		0.118327	1.40	6.03	-4.63	2.91	1.594	43

Multiple t-tests 4T1 vs 4T07 D21

CAF subset	Discovery?	P value	Mean1 4T1	Mean2 4T07	Difference	SE of difference	t ratio	df
S1		0.066789	1.66	0.76	0.90	0.47	1.890	36
S2	*	0.000003	1.97	0.39	1.58	0.29	5.503	36
S3		0.506516	0.29	0.19	0.09	0.14	0.671	36
S4		0.045289	0.46	0.09	0.38	0.18	2.074	36
S5		0.223138	0.02	0.05	-0.03	0.02	1.240	36
S6		0.011930	0.15	0.07	0.09	0.03	2.648	36
S7		0.211691	0.01	0.06	-0.05	0.04	1.272	36
S8		0.017864	0.21	0.04	0.17	0.07	2.482	36
S9		0.038304	1.50	0.71	0.79	0.37	2.151	36
S10		0.001291	2.97	0.53	2.44	0.70	3.491	36
S11		0.156885	0.59	0.33	0.26	0.18	1.446	36
S12	*	0.000914	1.35	0.25	1.10	0.31	3.614	36
S13		0.839347	0.00	0.00	0.00	0.00	0.204	36
S14		0.209145	0.03	0.02	0.01	0.01	1.279	36
S15		0.203697	0.01	0.01	-0.01	0.01	1.295	36
S16		0.038017	0.21	0.04	0.17	0.08	2.154	36
S17		0.243631	2.72	2.10	0.62	0.52	1.185	36
S18		0.003042	2.09	0.91	1.18	0.37	3.178	36
S19		0.955422	1.10	1.12	-0.02	0.35	0.056	36
S20		0.034774	1.17	0.66	0.51	0.23	2.194	36
S21	*	0.000303	0.38	1.17	-0.79	0.20	3.998	36
S22		0.164183	13.21	8.91	4.29	3.02	1.420	36
S23		0.020824	0.57	1.02	-0.45	0.19	2.417	36
S24		0.230951	11.35	8.95	2.40	1.97	1.219	36
S25		0.124650	2.02	1.13	0.89	0.56	1.572	36
S26		0.039129	0.64	0.29	0.35	0.16	2.141	36
S27		0.502708	1.92	2.45	-0.53	0.78	0.677	36
S28		0.686840	0.64	0.57	0.07	0.17	0.406	36
S29		0.167042	0.04	0.09	-0.06	0.04	1.410	36
S30		0.004817	0.43	0.23	0.21	0.07	3.005	36
S31		0.088126	0.37	1.06	-0.68	0.39	1.753	36
S32		0.669595	15.09	13.72	1.38	3.20	0.430	36
S33		0.853266	0.22	0.21	0.02	0.08	0.186	36
S34		0.028409	0.45	0.24	0.21	0.09	2.283	36
S35		0.214440	0.05	0.02	0.03	0.02	1.264	36
S36		0.095830	0.28	0.08	0.20	0.12	1.710	36
S37		0.375778	0.01	0.01	0.01	0.01	0.897	36
S38		0.053825	0.11	0.05	0.06	0.03	1.994	36
S39		0.531654	0.01	0.01	0.00	0.01	0.632	36
S40		0.026662	0.31	0.05	0.26	0.11	2.311	36
S41		0.104877	0.53	0.84	-0.31	0.19	1.664	36
S42		0.126384	1.79	0.99	0.81	0.52	1.565	36
S43		0.686055	0.25	0.20	0.04	0.10	0.407	36
S44		0.162772	1.13	0.62	0.51	0.36	1.425	36
S45		0.060755	0.01	0.02	-0.01	0.00	1.936	36
S46		0.001589	0.24	0.09	0.15	0.05	3.416	36
S47		0.500512	0.03	0.05	-0.02	0.02	0.681	36
S48		0.252844	1.06	0.59	0.48	0.41	1.162	36
S49		0.004597	0.31	0.58	-0.27	0.09	3.023	36
S50		0.227580	0.53	0.68	-0.15	0.12	1.228	36
S51		0.066808	0.13	0.31	-0.17	0.09	1.890	36
S52		0.067913	0.55	0.32	0.22	0.12	1.882	36
S53	*	0.000005	0.10	0.56	-0.46	0.09	5.354	36
S54		0.497700	6.19	7.66	-1.47	2.14	0.685	36
S55		0.019206	0.82	2.31	-1.49	0.61	2.452	36
S56		0.095990	14.55	16.88	-2.32	1.36	1.709	36
S57		0.402479	0.52	0.63	-0.12	0.14	0.847	36
S58		0.695238	0.50	0.58	-0.08	0.19	0.395	36
S59		0.170736	0.46	0.71	-0.26	0.18	1.398	36
S60		0.657369	0.69	0.62	0.08	0.17	0.447	36
S61	*	0.000009	0.07	0.80	-0.73	0.14	5.158	36
S62		0.478906	1.47	1.72	-0.25	0.35	0.716	36
S63	*	0.000849	1.45	13.69	-12.24	3.36	3.640	36
